# Direct Generation and Non-Hermitian Regulation of Energy-Time-Polarization-Hyper-Entangled Quadphotons

**DOI:** 10.3390/s25113425

**Published:** 2025-05-29

**Authors:** Rui Zhuang, Siqiang Zhang, Guobin Liu, Zhou Feng, Qingyu Chen, Sinong Liu, Yanpeng Zhang

**Affiliations:** Key Laboratory for Physical Electronics and Devices of the Ministry of Education, Shaanxi Key Lab of Information Photonic Technique, School of Electronic Science and Engineering, Xi’an Jiaotong University, Xi’an 710049, China; zhuangray@stu.xjtu.edu.cn (R.Z.); siqiangzhang@stu.xjtu.edu.cn (S.Z.); 2803036011@stu.xjtu.edu.cn (G.L.); second2fz@163.com (Z.F.); shefieldlannar@outlook.com (Q.C.); liu-sinong@stu.xjtu.edu.cn (S.L.)

**Keywords:** non-linear optics, atomic optics, quantum optics

## Abstract

Entangled multiphoton is an ideal resource for quantum information technology. Here, narrow-bandwidth hyper-entangled quadphoton is theoretically demonstrated by quantizing degenerate Zeeman sub states through spontaneous eight-wave mixing (EWM) in a hot ^85^Rb. Polarization-based energy-time entanglement (output) under multiple polarized dressings is presented in detail with uncorrelated photons and Raman scattering suppressed. High-dimensional entanglement is contrived by passive non-Hermitian characteristic, and EWM-based quadphoton is genuine quadphoton with quadripartite entanglement. High quadphoton production rate is achieved from co-action of four strong input fields, and electromagnetically induced transparency (EIT) slow light effect. Atomic passive non-Hermitian characteristic provides the system with acute coherent tunability around exceptional points (EPs). The results unveil multiple coherent channels (~8) inducing oscillations with multiple periods (~19) in quantum correlations, and high-dimensional (~8) four-body entangled quantum network (capacity ~65536). Coexistent hyper and high-dimensional entanglements facilitate high quantum information capacity. The system can be converted among three working states under regulating passive non-Hermitian characteristic via triple polarized dressing. The research provides a promising approach for applying hyper-entangled multiphoton to tunable quantum networks with high information capacity, whose multi-partite entanglement and multiple-degree-of-freedom properties help optimize the accuracy of quantum sensors.

## 1. Introduction

Nonclassical multiphoton plays key roles on quantum information technology such as quantum network, obtaining quantum light sources with high-dimensional entanglement characteristics and greater information capacity has been the focus of quantum information research to realize breakthroughs [1,2]. Biphoton is generated via spontaneous parametric down-conversion (SPDC) [3] and four-wave mixing (FWM) [4]. Triphoton is generated by cascaded nonlinear parameter processes [5,6,7], post-selection [8], six-wave mixing (SWM) [9], etc. Attention is focused on quadphoton owing to increasing quantum information capacity which can be promoted by hyper [10] and high-dimensional entanglements [11], thus optimizing quantum communications [12,13].

SPDC-based biphoton is criticized for short coherent time and wide bandwidth. FWM-based biphoton and SWM-based triphoton feebly promote information capacity. Post-selection-based quadphoton is essentially biphoton which presents low generation efficiency. Moreover, bare quadphoton difficultly maximizes information capacity for lacking assisted means of hyper and high-dimensional entanglements.

Much theoretical and experimental work has been done on biphoton [14,15], triphoton [16,17,18,19] and multiphoton [20] which suffers natural physical inequality among photons. Hyperentangled [10] and polarization-entangled [21] biphoton are experimentally generated. *x*^(5)^-based polarization-entangled [18] or high-dimensional energy-time-entangled [19] triphoton is theoretically realized. To our knowledge, there is no study on *x*^(7)^-based hyperentangled and high-dimensional entangled quadphoton. Besides, atomic non-Hermitian system is less studied [22] comparing with other systems [23]. There is yet no tunable high-dimensional entanglement produced via atomic non-Hermitian characteristic.

EWM is a bright method to directly produce genuine quadphoton. Such approach has been theoretically presented in our previous work [24,25,26]. In this single physical process of EWM based on a pure high order nonlinearity (*x*^(7)^), the co-action of four strong input fields as well as EIT slow light effect endows the generated quadphoton with a natural high generation rate. For this quadphoton process, coexistent hyper and high-dimensional entanglements can be originally constructed when the coherent polarized dressing is used to control the degenerate Zeeman sub states in the atomic energy levels. Based on this, it is likely to build tunable high-dimensional entanglement, with passively regulating the atomic non-Hermitian characteristic.

In this paper, we theoretically generate *x*^(7)^-based energy-time-polarization-hyper-entangled quadphotons in ^85^Rb hot atomic vapor. The quadphotons’ generation mainly utilizes the Zeeman sub-state of the atom. A spatially separated optical pumping is used to suppress uncorrelated photons. We evaluate conditioned three-photon correlation, quad-photon nonclassical characteristics and correlation. Polarization-based energy-time entanglement is presented in the hyper-entanglement. The utilization of single, double, and triple polarized dressing is employed in the quantization of Zeeman sub-states at the high dimensional level. The construction of tunable high-dimensional entanglement is facilitated by the passive non-Hermitian characteristic of triply dressed atoms. The results show multiple coherent channels inducing Rabbi oscillations accompanied by multiple periods in quantum correlations, and high-dimensional four-body entangled quantum network elements. *x*^(7)^-based genuine quadphoton with high production rate, directly generated in single step, is capable of quadripartite entanglement source for quantum information. Coexistent hyper and high-dimensional entanglements, and triply dressed adjustable non-Hermitian characteristic make the quadphoton become a potential candidate for tunable quantum networks with high information capacity.

## 2. Basic Theory and Simulation

### 2.1. Quadphoton with Energy-Time-Polarization Hyper-Entanglement

In order to generate the hyper-entanglement, we make use of the degenerate Zeeman sub states of each hyperfine energy level of ^85^Rb (D_1_ line (795 nm), D_2_ line (780 nm), 776 nm). The Zeeman states with $\Delta M_{F}=0$ are coupled by a linearly polarized pump field and three coupling fields. Among them, the linearly polarized pump field is represented by the horizontal polarization *H*_2_; the coupled fields are represented by two vertical polarizations *V*_1_, *V*_3_ and one horizontal polarization *H*_4_. Given the fact that linearly polarized light can be decomposed into circularly polarized light, we can obtain the following relationships: $\left|V_{1}\right\rangle =i(\left|\sigma_{1}^{+}\right\rangle -\left|\sigma_{1}^{-}\right\rangle )/\sqrt{2}$, $\left|H_{2}\right\rangle =(\left|\sigma_{2}^{+}\right\rangle +\left|\sigma_{2}^{-}\right\rangle )/\sqrt{2}$, $\left|V_{3}\right\rangle =i(\left|\sigma_{3}^{+}\right\rangle -\left|\sigma_{3}^{-}\right\rangle )/\sqrt{2}$ and $\left|H_{4}\right\rangle =(\left|\sigma_{4}^{+}\right\rangle +\left|\sigma_{4}^{-}\right\rangle )/\sqrt{2}$. It is important to note that two distinct types of circularly polarized modes exist, which correspond to the incidence of $\left|\sigma_{1}^{+}\right\rangle \left|\sigma_{2}^{+}\right\rangle \left|\sigma_{3}^{-}\right\rangle \left|\sigma_{4}^{-}\right\rangle /4$ and $\left|\sigma_{1}^{-}\right\rangle \left|\sigma_{2}^{-}\right\rangle \left|\sigma_{3}^{+}\right\rangle \left|\sigma_{4}^{+}\right\rangle /4$, respectively. $\sigma^{+}$ and $\sigma^{-}$ are right circular polarization and left circular polarization, respectively.

**Figure 1:**
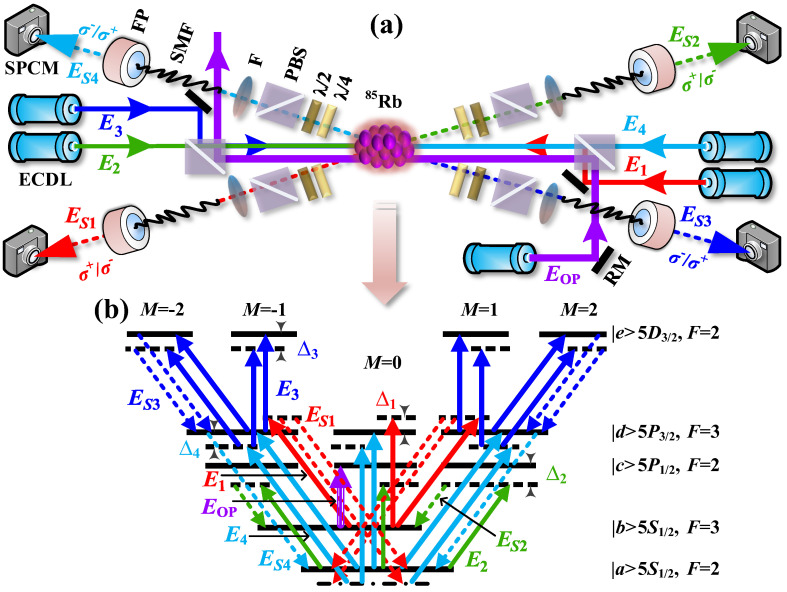
The following scheme is proposed for the generation of hyperentangled quadphotons in a five-level atomic system. (**a**) Experimental setup. The generation of quadphotons is detected by four equidistant single-photon counting modules (SPCMs) situated at the core of an ^85^Rb vapor cell. The experimental setup includes a PBS (polarization beam splitter), an RM (reflective mirror), an SMF (single-mode fiber), a FP (Fabry-Perot cavity), a F (filter), an SPCM (single-photon counting module), and an ECDL (external cavity diode laser). (**b**) Energy-level diagram with two possible polarization configurations for the spontaneously emitted quadphotons S1-4 from ^85^Rb (D_1_ line (795 nm), D_2_ line (780 nm), 776 nm) five-level atomic system. A strong optical-pumping laser ***E***_op_ is exploited to optically pump atoms from |*b*> to |*a*>, to suppress on-resonance Raman scattering of coupling beams [14].

The hyperfine energy levels are adopted as shown in Figure 1b, and considering that the polarizations of the S1-4 photons respectively follow the corresponding coupling and pump field in each incidence case, there are two kinds of coherent channels corresponding to the spontaneous processes defined as $\left|\sigma_{1}^{+}\right\rangle \left|\sigma_{2}^{+}\right\rangle \left|\sigma_{3}^{-}\right\rangle \left|\sigma_{4}^{-}\right\rangle \to \left|\sigma_{S1}^{+}\right\rangle \left|\sigma_{S2}^{+}\right\rangle \left|\sigma_{S3}^{-}\right\rangle \left|\sigma_{S4}^{-}\right\rangle$ and $\left|\sigma_{1}^{-}\right\rangle \left|\sigma_{2}^{-}\right\rangle \left|\sigma_{3}^{+}\right\rangle \left|\sigma_{4}^{+}\right\rangle \to \left|\sigma_{S1}^{-}\right\rangle \left|\sigma_{S2}^{-}\right\rangle \left|\sigma_{S3}^{+}\right\rangle \left|\sigma_{S4}^{+}\right\rangle$. Thus, it allows two possible circularly polarized configurations $\left|\sigma_{S1}^{+}\right\rangle \left|\sigma_{S2}^{+}\right\rangle \left|\sigma_{S3}^{-}\right\rangle \left|\sigma_{S4}^{-}\right\rangle /4$ and $\left|\sigma_{S1}^{-}\right\rangle \left|\sigma_{S2}^{-}\right\rangle \left|\sigma_{S3}^{+}\right\rangle \left|\sigma_{S4}^{+}\right\rangle /4$. Their output photons can be collected at the detectors.

Consequently, we can model energy-time-polarization hyper-entanglement by EWM process as

(1) $$\left.|\Psi_{S1-4}^{M}\right\rangle =\psi ⨂\left.|\varphi^{M}\right\rangle$$

The entangled four-photons produced by the EWM process are denoted as S1-4 photons and measured by each of the four detectors. The obtained hyper-entangled quantum states are described as follows:

(2) $$\left.|\Psi_{S1-4}^{M}\right\rangle =\left.|\Psi_{S1-4}^{1M}\right\rangle +\left.|\Psi_{S1-4}^{2M}\right\rangle$$

where $\left|\Psi_{S1-4}^{1M}\right\rangle =\psi (t_{S1},t_{S2},t_{S3},t_{S4})\exp(-i\varpi_{S1}t_{S1}-i\varpi_{S2}t_{S2}-i\varpi_{S3}t_{S3}-i\varpi_{S4}t_{S4})\left|\sigma_{S1}^{+}\sigma_{S2}^{+}\sigma_{S3}^{-}\sigma_{S4}^{-}\right\rangle /\sqrt{2}$,$\left|\Psi_{S1-4}^{2M}\right\rangle =\psi (t_{S1},t_{S2},t_{S3},t_{S4})\exp(-i\varpi_{S1}t_{S1}-i\varpi_{S2}t_{S2}-i\varpi_{S3}t_{S3}-i\varpi_{S4}t_{S4})\left|\sigma_{S1}^{-}\sigma_{S2}^{-}\sigma_{S3}^{+}\sigma_{S4}^{+}\right\rangle /\sqrt{2}$. $\left|\Psi_{S1-4}^{1M}\right\rangle$ and $\left|\Psi_{S1-4}^{2M}\right\rangle$ can be viewed as polarization-based energy-time entanglement states, which have similar entanglement performance, here we mainly discuss the former state and relevant spontaneous process in detail.

### 2.2. The Schematic of Quadphoton Generation

A simplified experimental setup of quadphoton generation is illustrated in Figure 1a, where the occurrence of EWM process happens through ^85^Rb atomic vapor. With identical five-level atoms initially prepared in their ground level |*a*> (see Figure 1b), the atomic vapor is confined within a long and thin cylindrical volume. The power of pump beam ***E***_2_ (horizontal polarization, ~795 nm, frequency *ω*_2_, wave vector ***k***_2_, Rabi frequency *G*_2_) is about several milliwatts, where $G_{i}=\mu_{\mathrm{ij}}E_{i}/\hbar$, $\mu_{\mathrm{ij}}$ is dipole moment between energy levels |i> and |j>. The power of coupling beams ***E***_1_ (vertical polarization, ~780 nm, *ω*_1_, ***k***_1_, *G*_1_), ***E***_3_ (vertical polarization, ~776 nm, *ω*_3_, ***k***_3_, *G*_3_) and ***E***_4_ (horizontal polarization, ~780 nm, *ω*_4_, ***k***_4_, *G*_4_) is over 10 milliwatts. The power of optical-pumping beam ***E***_op_ (vertical polarization, ~795 nm) is over 30 milliwatts. ***E***_1_, ***E***_4_ and ***E***_op_ counter-propagate with ***E***_2_. ***E***_3_ propagates in ***E***_2_ direction. It should be noted that ***E***_op_ is aligned parallel to ***E***_1–4_ without overlapping. The schematic of quadphotons generated via a five-level atomic system is shown in Figure 1b. The role of ***E***_2_ is to complete the process of atomic leaps |*a*>→|*c*> with detuning denoted as ∆_2_. Based on the above properties, this process significantly suppresses the quantum atomic noise and allows the atomic population to dominate in the ground state |*a*> [15] For the other three coupling beams ***E***_i_ (i = 1, 3, 4), the detuning is denoted as $\Delta_{i}$ (i = 1, 3, 4), corresponding to the atomic transitions |*b*>→|*d*>, |*d*>→|*e*>, and |*a*>→|*d*>, respectively. Here, $\Delta_{i}=\omega_{\mathrm{ij}}-\omega_{i}$ is detuning defined as the frequency difference between the resonant transition frequency *ω*_ij_ and laser frequency *ω*_i_ of the field ***E***_i_. The coupling beam ***E***_1_ has been demonstrated to facilitate the EWM nonlinear process and to open the transparency window for S1 photons through the slow-light effect [19]. ***E***_OP_ is on resonance to the atomic transition |*b*>→|*c*>. The EWM process meets the energy conservation *ћω*_1_ + *ћω*_2_ + *ћω*_3_ + *ћω*_4_ = *ћω_S_*_1_ + *ћω_S_*_2_ + *ћω_S_*_3_ + *ћω_S_*_4_, where *ω_S_*_i_ = *ϖ_S_*_i_ + *δ*_i_. Then, with the phase-matching condition *ћ**k***_1_ + *ћ**k***_2_ + *ћ**k***_3_ + *ћ**k***_4_ = *ћ**k**_S_*_1_ + *ћ**k**_S_*_2_ + *ћ**k**_S_*_3_ + *ћ**k**_S_*_4_ and low-gain limit, the EWM process will occur spontaneously, which could generate entangled quadphotons S1–4 [24,25,26].

In the interaction picture, the effective interaction Hamiltonian of EWM process can be expressed as (ignoring the reflections from the systemic surfaces and using the rotating-wave approximation)

(3) $$\begin{array}{l} {\hat{H}}_{I}=\varepsilon_{0}\int_{V}d^{4}r\chi^{\left(7\right)}E_{1}^{\left(+\right)}E_{2}^{\left(+\right)}E_{3}^{\left(+\right)}E_{4}^{\left(+\right)}E_{S4}^{\left(-\right)}E_{S3}^{\left(-\right)}E_{S2}^{\left(-\right)}E_{S1}^{\left(-\right)}+H.c.\\ \;=P_{1}\int d\omega_{S1}\int d\omega_{S2}\int d\omega_{S3}\int d\omega_{S4}\kappa \Phi (\Delta kL){\hat{a}}_{S1}^{†}{\hat{a}}_{S2}^{†}{\hat{a}}_{S3}^{†}{\hat{a}}_{S4}^{†}e^{-i\Delta \omega t}\\ \;+\;H.c. \end{array}$$

where vacuum permittivity is denoted by $\varepsilon_{0}$, volume illuminated by input beams ***E***_1–4_ together is denoted by *V*, seventh-order nonlinear susceptibility is denoted by *χ*^(7)^, positive-frequency part of input beam ***E***_i_ is denoted by $E_{i}^{\left(+\right)}$, quantum field amplitude of Si photon is denoted by $E_{Si}^{\left(-\right)}$, and Hermitian conjugate is denoted by *H*.*c*. $P_{1}=i\hbar^{2}/\pi^{2}\varepsilon_{0}^{2}\iota^{2}$ is a constant, and ι is the cross-section area of single mode; The nonlinear parametric coupling coefficient is represented by $\kappa =-i(\varpi_{S1}\varpi_{S2}\varpi_{S3}\varpi_{S4}{)}^{1/2}\chi^{\left(7\right)}(\omega_{S1},\omega_{S2},\omega_{S3},\omega_{S4})E_{1}E_{2}E_{3}E_{4}/c^{2}$. The central frequency of a Si photon is denoted by *ϖ_S_*_i_. The speed of light in a vacuum, c, is also included in this equation. The electric field intensity $E_{i}$ is expressed as $E_{i}=i(\hbar \omega_{i}/2\varepsilon_{0}V_{q}{)}^{1/2}n_{i}$. The term *V*_q_ signifies the quantization volume. The longitudinal detuning function, denoted by Φ(ΔkL)=sinc(ΔkL/2)exp⁡(−iΔkL/2), serves to determine the natural spectral width of quadphoton. $\Delta k=k_{S1}+k_{S2}+k_{S3}+k_{S4}-k_{1}-k_{2}-k_{3}-k_{4}$ is the phase mismatching of quadphoton, the phase-match condition holds perfectly when Δ*k* is equal to 0, and *k*_i_ is wavenumber of field; ${\hat{a}}_{Si}^{†}$ is the annihilation operator of the output mode for Si photon; Δ*ω* = *ω*_1_ + *ω*_2_ + *ω*_3_ + *ω*_4_ − *ω_S_*_1_ − *ω_S_*_2_ − *ω_S_*_3_ − *ω_S_*_4_.

Considering the first-order perturbation in the interaction picture, the photon state at the output surface is approximately the linear superposition of the vacuum state |0> and |*ψ*>. Given the unobservability of the vacuum state, it is set to one side and ignored in further analysis. The state |*ψ*> of quadphoton can be expressed as

(4) $$\begin{array}{l} \left.|\psi \right\rangle =\frac{-i}{\hbar }\int_{-\infty }^{+\infty }dt_{S1}\int_{-\infty }^{+\infty }dt_{S2}\int_{-\infty }^{+\infty }dt_{S3}\int_{-\infty }^{+\infty }dt_{S4}{\hat{H}}_{I}\left.|0\right\rangle \\ =\int d\omega_{S1}\int d\omega_{S2}\int d\omega_{S3}\int d\omega_{S4}\kappa \Phi \left(\Delta kL\right){\hat{a}}_{S1}^{†}{\hat{a}}_{S2}^{†}{\hat{a}}_{S3}^{†}{\hat{a}}_{S4}^{†}\delta \left(\Delta \omega \right)\left.|0\right\rangle \\ =\int d\omega_{S1}\int d\omega_{S2}\int d\omega_{S3}\int d\omega_{S4}\kappa \Phi \left(\Delta kL\right){\hat{a}}_{S1}^{†}{\hat{a}}_{S2}^{†}{\hat{a}}_{S3}^{†}{\hat{a}}_{S4}^{†}\left.|0\right\rangle \end{array}$$

Combining with Equations (3) and (4) [25], exp(−*i*Δ*ωt*) becomes 2*πδ*(Δ*ω*), which indicates the energy conservation of EWM process and leads to the frequency entanglement of the quadphoton state. As demonstrated in Equation (4), the quadphoton state exhibits entanglement in both frequency and wave number. This entanglement is illustrated by $\kappa =\kappa (\omega_{S1},\omega_{S2},\omega_{S3},\Delta \omega +\omega_{S4})$ in the frequency domain, which is due to energy conservation. The wave number entanglement, denoted by Φ(ΔkL), is an inherent property of the system under consideration. It is not possible to decompose Φ(ΔkL) into four independent functions, each containing only *k_S_*_1–4_. In the general noncollinear case, wave-number entanglement exerts a significant influence on the spatial correlation of quadphotons.

In order to engage in discourse on the optical properties of quadphotons generated via a five-level system, it is necessary to consider the measurement of the average quad-photon coincidence counting rate (Rcc). Rcc is a significant characterization metric of energy-time-entangled multiphotons [5,7,15]. From Rcc, we are able to obtain periods of oscillation, coherent time, and other crucial parameters. We suppose the detectors SPCM1-4 detect photons with frequency *ω_S_*_1–4_, respectively. Assuming perfect detection efficiency, *Rcc* is defined by

(5) $$Rcc\left(t_{S1},t_{S2},t_{S3},t_{S4}\right)=\underset{T\to \infty }{\lim}\frac{1}{T}\int_{0}^{T}dt_{S1}\int_{0}^{T}dt_{S2}\int_{0}^{T}dt_{S3}\int_{0}^{T}dt_{S4}G^{\left(4\right)}\left(t_{S1},t_{S2},t_{S3},t_{S4}\right)$$

The fourth-order correlation function (CF), denoted *G*^(4)^(*t_S_*_1_,*t_S_*_2_,*t_S_*_3_,*t_S_*_4_), is the probability of four correlated coincidence events being detected jointly. According to Glauber’s theory, it can be written as:

(6) $$\begin{array}{ll} G^{\left(4\right)}\left(t_{S1},t_{S2},t_{S3},t_{S4}\right) & =\left|\left\langle \psi |\right.E_{S1}^{\left(-\right)}E_{S2}^{\left(-\right)}E_{S3}^{\left(-\right)}E_{S4}^{\left(-\right)}E_{S4}^{\left(+\right)}E_{S3}^{\left(+\right)}E_{S2}^{\left(+\right)}E_{S1}^{\left(+\right)}\left.|\psi \right\rangle \right|\\ & ={\left|\left\langle 0|\right.E_{S4}^{\left(+\right)}E_{S3}^{\left(+\right)}E_{S2}^{\left(+\right)}E_{S1}^{\left(+\right)}\left.|\psi \right\rangle \right|}^{2}={\left|B\left(t_{S1},t_{S2},t_{S3},t_{S4}\right)\right|}^{2} \end{array}$$

where *t_S_*_i_ = *t_S_*_i0_ − *r_S_*_i_/*c*, with *r_S_*_i_ representing the length of Si’s optical path from the output surface of the medium to the detector. For the sake of simplicity, it is assumed that *r_S_*_1_ = *r_S_*_2_ = *r_S_*_3_ = *r_S_*_4_. $B\left(t_{S1},t_{S2},t_{S3},t_{S4}\right)$ is the amplitude of quadphoton, which can be expressed as

(7) $$B\left(t_{S1},t_{S2},t_{S3},t_{S4}\right)=P_{2}\int d\omega_{S1}\int d\omega_{S2}\int d\omega_{S3}\int d\omega_{S4}\kappa \Phi (\Delta kL)e^{-i\sum_{i=1}^{4}\omega_{Si}t_{Si}}$$

where *P*_2_ is a constant which absorbs all the constants and slowly varying terms. By using Equation (7), one can obtain

(8) $$B\left(\tau_{1},\tau_{2},\tau_{3}\right)=\zeta (\tau_{1},\tau_{2},\tau_{3})e^{-i(\omega_{1}+\omega_{2}+\omega_{3}+\omega_{4})t_{S4}}$$

(9) $$\zeta \left(\tau_{1},\tau_{2},\tau_{3}\right)=\frac{L}{2\pi }\int d\delta_{1}\int d\delta_{2}\int d\delta_{3}\kappa (\delta_{1},\delta_{2},\delta_{3})\Phi (\delta_{1},\delta_{2},\delta_{3})e^{-i(\delta_{1}\tau_{1}+\delta_{2}\tau_{2}+\delta_{3}\tau_{3})}$$

where *τ*_1_ = *t_S_*_1_ − *t_S_*_4_, *τ*_2_ = *t_S_*_2_ − *t_S_*_4_ and *τ*_3_ = *t_S_*_3_ − *t_S_*_4_ are the relative time. The S1-3 photons are used differently from the S4 photon, with the former being coincidence counting stop photons and the latter being coincidence counting trigger photon. In time domain, the wave function of quadphoton is a convolution of *κ* and Φ as $\zeta (\tau_{1},\tau_{2},\tau_{3})=L[\tilde{\kappa }(\tau_{1},\tau_{2},\tau_{3})*\tilde{\Phi }(\tau_{1},\tau_{2},\tau_{3})]$. Where $\tilde{\kappa }(\tau_{1},\tau_{2},\tau_{3})=\int d\delta_{1}\int d\delta_{2}\int d\delta_{3}\kappa (\delta_{1},\delta_{2},\delta_{3})\exp(-i(\delta_{1}\tau_{1}+\delta_{2}\tau_{2}+\delta_{3}\tau_{3}))/(2\pi )$ and $\tilde{\Phi }(\tau_{1},\tau_{2},\tau_{3})=\int d\delta_{1}\int d\delta_{2}\int d\delta_{3}\Phi (\delta_{1},\delta_{2},\delta_{3})\exp(-i(\delta_{1}\tau_{1}+\delta_{2}\tau_{2}+\delta_{3}\tau_{3}))/(2\pi )$.

### 2.3. Evaluation on the Correlations of Conditioned Three-Photon and Quadphoton

The correlation of conditioned three-photon is evaluated as Supplement S1. Checking the violation of Cauchy-Schwarz inequality (*C*.*S*.) is usually used to estimate the nonclassical behavior of multiphoton state. According to the inequality of ${\left\langle uvwl\right\rangle }^{2}\leq \left\langle u^{2}\right\rangle \left\langle v^{2}\right\rangle \left\langle w^{2}\right\rangle \left\langle l^{2}\right\rangle$ for quadphoton, their correlated properties can be estimated through Equation (10).

(10) $$C.S.=\frac{{\left[g_{S1-4}^{\left(4\right)}(\tau_{1},\tau_{2},\tau_{3})=\right]}^{2}}{\prod_{i=1}^{4}g_{Si,Si}^{\left(2\right)}(0)}\leq 1$$

where $g_{S1-4}^{\left(4\right)}(\tau_{1},\tau_{2},\tau_{3})=1+g_{S1-4}^{\left(4\right)}(\tau_{1},\tau_{2},\tau_{3})/(R_{S1}R_{S2}R_{S3}R_{S4})$ is the normalized cross-CF of quadphoton; $g_{Si,Si}^{\left(2\right)}(0)=1+G_{Si,Si}^{\left(2\right)}/R_{Si}^{2}\leq 2$ is the normalized auto-CF of Si photon and can be obtained via a beam splitter. When the obtained C.S. is larger than 1, Cauchy-Schwarz inequality is violated, which indicates a strong nonclassical correlation in the quadphoton state.

The noise accompanying generation of quadphoton is mainly because of the third and fifth-order nonlinearity. Therefore, the actual *Rcc* of quadphoton can be written as

(11) $$\begin{array}{ll} {Rcc}_{M}\left(\tau_{1},\tau_{2},\tau_{3}\right) & =\underset{T\to \infty }{\lim}\frac{1}{T}\int_{0}^{T}dt_{S1}\int_{0}^{T}dt_{S2}\int_{0}^{T}dt_{S3}[G_{S1-4}^{\left(4\right)}\left(\tau_{1},\tau_{2},\tau_{3}\right)+\sum_{i=1}^{4}G_{i}^{\left(3\right)}\\ & +\sum_{i=1}^{9}G_{i}^{\left(2\right)}+\prod_{i=1}^{4}R_{Si}] \end{array}$$

where $G_{1}^{\left(3\right)}$ is CF of SWM1 triphotons (***k***_1_ + ***k***_2_ + ***k***_3_ = ***k****_S_*_1_ + ***k****_S_*_2_ + ***k****_S_*_3_); $G_{2}^{\left(3\right)}$ is CF of SWM2 triphotons (***k***_1_ + ***k***_2_ + ***k***_4_ = ***k****_S_*_1_ + ***k****_S_*_2_ + ***k****_S_*_4_); $G_{3}^{\left(3\right)}$ is CF of SWM3 triphotons (***k***_1_ + ***k***_3_ + ***k***_4_ = ***k****_S_*_1_ + ***k****_S_*_3_ + ***k****_S_*_4_); $G_{4}^{\left(3\right)}$ is CF of SWM4 triphotons (***k***_2_ + ***k***_3_ + ***k***_4_ = ***k****_S_*_2_ + ***k****_S_*_3_ + ***k****_S_*_4_); $G_{1}^{\left(2\right)}$ is CF of phase conjugate FWM1 (PCFWM1) biphotons (***k***_1_ + ***k***_2_ = ***k****_S_*_1_ + ***k****_S_*_2_); $G_{2}^{\left(2\right)}$ is CF of PCFWM2 biphotons (***k***_1_ + ***k***_3_ = ***k****_S_*_3_ + ***k****_S_*_4_); $G_{3}^{\left(2\right)}$ is CF of self-diffraction FWM3 (SDFWM3) biphotons (***k***_1_ + ***k***_4_ = ***k****_S_*_1_ + ***k****_S_*_4_); $G_{4}^{\left(2\right)}$ is CF of PCFWM4 biphotons (***k***_2_ + ***k***_4_ = ***k****_S_*_1_ + ***k****_S_*_2_); $G_{5}^{\left(2\right)}$ is CF of PCFWM5 biphotons (***k***_3_ + ***k***_4_ = ***k****_S_*_3_ + ***k****_S_*_4_); $G_{6}^{\left(2\right)}$ is CF of SDFWM6 biphotons (2***k***_1_ = ***k****_S_*_1_ + ***k****_aS_*_1_); $G_{7}^{\left(2\right)}$ is CF of SDFWM7 biphotons (2***k***_2_ = ***k****_S_*_2_ + ***k****_aS2_*); $G_{8}^{\left(2\right)}$ is CF of SDFWM8 biphotons (2***k***_3_ = ***k****_S_*_3_ + ***k****_aS3_*); $G_{9}^{\left(2\right)}$ is CF of SDFWM9 biphotons (2***k***_4_ = ***k****_S_*_4_ + ***k****_aS_*_4_); *R_S_*_1–4_ are the counting rates of uncorrelated single photons [27], which originate from S1-4 photons of SDFWM6-9 biphotons, respectively. Because the central frequency difference between aS1 and S4, aS2 and S3, aS3 and S2, aS4 and S1 photons, is more than 3 GHz, the noise of aS1-4 photons is filtered out by filters and narrowband etalon FPs before being detected by the SPCMs as illustrated in Figure 1a. In addition, triphotons of SWM1(2) and biphotons of PCFWM1(5) will pass through filters and FPs. However, one of triphotons of SWM3(4) will be filtered out by filter and FP, and another two photons will pass through another two filters and FPs; one of biphotons of PCFWM2(SDFWM3, PCFWM4) will be filtered out by filter and FP, and another photon will pass through another filter and FP. As a result, the actual *Rcc* of quadphoton (after filtering) can be rewritten as

(12) $$\begin{array}{ll} {Rcc}_{M}\left(\tau_{1},\tau_{2},\tau_{3}\right) & =\underset{T\to \infty }{\lim}\frac{1}{T}\int_{0}^{T}dt_{S1}\int_{0}^{T}dt_{S2}\int_{0}^{T}dt_{S3}[G_{S1-4}^{\left(4\right)}\left(\tau_{1},\tau_{2},\tau_{3}\right)+G_{1}^{\left(3\right)}+G_{2}^{\left(3\right)}\\ & +\;G_{1}^{\left(2\right)}+G_{5}^{\left(2\right)}+{G^{\prime }}_{S3,S4}^{\left(2\right)}+{G^{\prime \prime }}_{S3,S4}^{\left(2\right)}+\prod_{i=1}^{4}R_{Si}] \end{array}$$

where ${G^{\prime }}_{S3,S4}^{\left(2\right)}$ and ${G^{\prime \prime }}_{S3,S4}^{\left(2\right)}$ is cross-CF of filtered triphotons of SWM3 and SWM4, respectively. *R*′*_S_*_1–4_ are the counting rates of uncorrelated single photons. *R*′*_S_*_1_ originates from S1 photons of biphotons of SDFWM3 and SDFWM6; *R*′*_S_*_2_ originates from S2 photons of biphotons of PCFWM4 and SDFWM7; *R*′*_S_*_3_ originates from S3 photons of biphotons of PCFWM2 and SDFWM8; *R*′*_S_*_4_ originates from S4 photons of biphotons of SDFWM3 and SDFWM9. Consequently, the accidental coincidence counting of quadphoton coincidence counting in actual measurements is caused by SWM1, SWM2, PCFWM1, PCFWM5, ${G^{\prime }}_{S3,S4}^{\left(2\right)}$, ${G^{\prime \prime }}_{S3,S4}^{\left(2\right)}$, uncorrelated single photons, and dark count of SPCMs, which constitute the background of coincidence counting of quadphoton. In addition to using filters, FPs and optical pumping as depicted in Figure 1, the accidental coincidence counting can be effectively reduced by finely placing SPCMs at appropriate angles which satisfy the phase-matching condition of EWM process as soon as possible, however, dissatisfy the phase-matching conditions of SWM and FWM processes.

### 2.4. Optical Response and Coincidence Counts of Quadphoton

As in Equation (7), the amplitude of quadphoton is doubly determined by the nonlinear coefficient κ and longitudinal detuning function Φ(ΔkL). In our case, Rabi frequency $\Omega_{eM}$ and linewidth $\Gamma_{eM}$ are quite smaller than the phase-matching bandwidth $\Delta \omega_{gM}$ and EIT bandwidth $\Delta \omega_{trM}$, Φ(ΔkL) approximates one [19]. The wave function of quadphoton can be expressed as $\zeta (\tau_{1},\tau_{2},\tau_{3})\approx L\tilde{\kappa }(\tau_{1},\tau_{2},\tau_{3})$. Consequently, one simply considers the nonlinear optical response.

**Figure 2:**
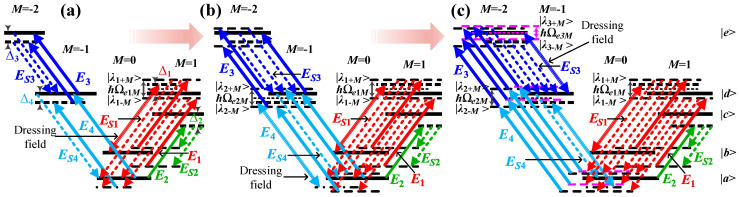
Schemes for producing quadphotons with polarization-based energy-time entanglement of multi-channel EWM in five-level atomic system. (**a**–**c**) Evolution of coherent channels whose energy is conservative from the ^85^Rb atomic energy-level diagram by EWM. (**a**) The model under consideration comprises four channels, each characterized by four-dimensional quantized energy levels and a single circularly polarized dressing field. (**b**) The model under consideration comprises six channels, each characterized by six-dimensional quantized energy levels and double circularly polarized dressing fields. (**c**) eight channels in eight-dimensional quantized energy-levels with three circularly polarized dressing fields.

Figure 2a–c show the rubidium atomic polarized energy-level diagrams of EWM in the “dressed-state” picture with multiple circularly polarized dressing fields. In Figure 2a, single circularly polarized dressing field ***E***_1_ is introduced to generate multiple coherent channels. The energy level |*d*, *M* = 1⟩ is separated into energy levels |*λ*_1±*M*_⟩ by the dressing effect of ***E***_1_. Consequently, the S1 photon acquires four dressed states: $\hbar (\varpi_{S1M}-\Delta_{4}-a_{\pm }+i\Gamma_{41M}-i\Gamma_{e1M})$ and $\hbar (\varpi_{S1M}-\Delta_{3}-\Delta_{4}-a_{\pm }+i\Gamma_{51M}-i\Gamma_{e1M})$. The S2 photon, in turn, has two dressed states: $\hbar (\varpi_{S2M}+a_{\pm }+i\Gamma_{e1M})$. The S3 photon has two states, $\hbar \varpi_{S3M}$ and $\hbar (\varpi_{S3M}+\Delta_{3}-i\Gamma_{51M}+i\Gamma_{41M})$, and the S4 photon has one single state, $\hbar (\varpi_{S4M}+\Delta_{4}-i\Gamma_{41M})$, which can be derived from Equation (S6). The collective output states and their corresponding input states coalesce to form four distinct polarization-based coherent channels, a phenomenon that is governed by the principle of energy conservation. In Figure 2b, circularly polarized dressing field ***E***_4_ with more power over Figure 2a is further added to generate more coherent channels. The energy level |*d*, *M* = −1⟩ is separated into energy levels |*λ*_2±*M*_⟩ by the dressing effect of ***E***_4_. Consequently, the S1 photon has six dressed states: $\hbar (\varpi_{S1M}+b_{\pm }-a_{\pm }+i\Gamma_{e2M}-i\Gamma_{e1M})$ and $\hbar (\varpi_{S1M}-\Delta_{3}-\Delta_{4}-a_{\pm }+i\Gamma_{51M}-i\Gamma_{e1M})$. The S2 photon has two dressed states: $\hbar (\varpi_{S2M}+a_{\pm }+i\Gamma_{e1M})$. The S3 photon has two dressed states: $\hbar (\varpi_{S3M}-b_{\pm }-\Delta_{4}-i\Gamma_{e2M}+i\Gamma_{41M})$ and one bare state $\hbar (\varpi_{S3M}+\Delta_{3}-i\Gamma_{51M}+i\Gamma_{41M})$. In the case of the S4 photon, a single bare state, $\hbar (\varpi_{S4M}+\Delta_{4}-i\Gamma_{41M})$, can be derived from Equation (S7). The collective output states and their corresponding input states coalesce to comprise six polarization-based coherent channels, a phenomenon that is governed by the principle of energy conservation. In Figure 2c, we introduce the third circularly polarized dressing field ***E***_3_ with more power over Figure 2b. In this case, the energy level |*e*, *M* = –2⟩ is divided into energy levels |*λ*_3±*M*_⟩ due to the dressing effect of ***E***_3_. Consequently, the S1 photon possesses eight dressed states: $\hbar (\varpi_{S1M}+b_{\pm }-a_{\pm }+i\Gamma_{e2M}-i\Gamma_{e1M})$ and $\hbar (\varpi_{S1M}+c_{\pm }-a_{\pm }+i\Gamma_{e3M}-i\Gamma_{e1M})$. The S2 photon has two dressed states, $\hbar (\varpi_{S2M}+a_{\pm }+i\Gamma_{e1M})$, and the S3 photon has four dressed states, $\hbar (\varpi_{S3M}-b_{\pm }-\Delta_{4}-i\Gamma_{e2M}+i\Gamma_{41M})$ and $\hbar (\varpi_{S3M}-c_{\pm }-\Delta_{4}-i\Gamma_{e3M}+i\Gamma_{41M})$, and S4 photon has one bare state $\hbar (\varpi_{S4M}+\Delta_{4}-i\Gamma_{41M})$, which are derived from Equation (S8). Similarly, it can form eight polarization-based coherent channels with energy conservation. Where $a_{\pm }=(-\Delta_{1}\pm \Omega_{e1M})/2$, $\Gamma_{e1M}=(\Gamma_{41M}+\Gamma_{21M})/2$; $b_{\pm }=(-\Delta_{4}\pm \Omega_{e2M})/2$, $\Gamma_{e2M}=(\Gamma_{41M}+\Gamma_{11M})/2$; $c_{\pm }=(-\Delta_{3}-2\Delta_{4}\pm \Omega_{e3M})/2$, $\Gamma_{e3M}=(\Gamma_{41M}+\Gamma_{51M})/2$; $\Omega_{e1M}=\sqrt{{\Omega_{e1M}}^{\prime }}$, ${\Omega_{e1M}}^{\prime }=\Delta_{1}^{2}+4[(\frac{\sqrt{5}}{6}G_{1M}{)}^{2}(2\mathrm{co}s^{2}\theta \sin^{2}\theta )]+4\Gamma_{41M}\Gamma_{21M}$, $\Omega_{e2M}=\sqrt{\Omega_{e2M}\prime }$, ${\Omega_{e2M}}^{\prime }=\Delta_{4}^{2}+4[(\frac{2}{3}\sqrt{\frac{1}{5}}G_{4M}{)}^{2}(2\mathrm{co}s^{2}\theta \sin^{2}\theta )]+4\Gamma_{41M}\Gamma_{11M}$, $\Omega_{e3M}=\sqrt{{\Omega_{e3M}}^{\prime }}$, ${\Omega_{e3M}}^{\prime }=(\Delta_{3}+2\Delta_{4}{)}^{2}+4[-\Delta_{4}(\Delta_{3}+\Delta_{4})+\Gamma_{41M}\Gamma_{51M}+(\frac{8}{15}\sqrt{\frac{2}{21}}G_{3M}{)}^{2}(2\mathrm{co}s^{2}\theta \sin^{2}\theta )]$, θ=π/4.

The perturbation chain is a suitable approach to directly present the relevant physical picture for demonstrating a multi-wave mixing process [28]. From Figure 2a, we can get the polarized perturbation chains as Equations (S4) and (S5). According to the polarized perturbation chains, the seventh-order nonlinear susceptibilities for the generated fields with single dressing (***E***_1_) (*SM*), double dressing (***E***_1_ and ***E***_4_) (*DM*) and triple dressing (***E***_1_, ***E***_4_ and ***E***_3_) (*TM*) are defined in Equations (S6)–(S8), respectively. According to Equations (S6)–(S8), the resonant positions and line widths of S1-4 photons are as shown in Table S1.

**Figure 3:**
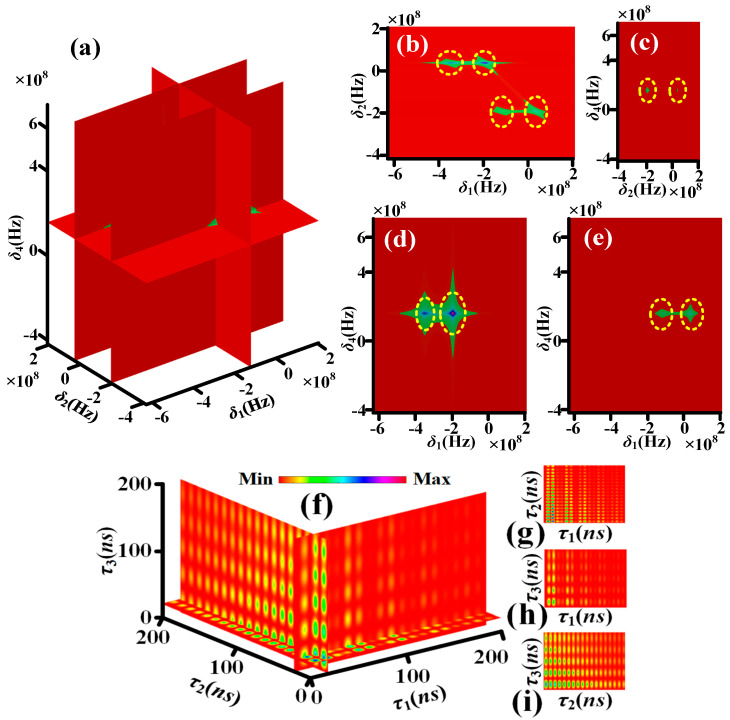
(**a**) The seventh-order nonlinear susceptibility $\left|\chi_{S4SM}^{\left(7\right)}\right|$ with single circularly polarized dressing field. (**b**) The nonlinear susceptibility in the flat $\delta_{1}-\delta_{2}$ of (**a**) with Re⁡(δ4)=Δ4. (**c**) The nonlinear susceptibility in the flat $\delta_{2}-\delta_{4}$ of (a) with Re⁡(δ1)=−Δ3−Δ4−a−. (**d**) The nonlinear susceptibility in the flat $\delta_{1}-\delta_{4}$ of (a) with Re⁡(δ2)=a+. (**e**) The nonlinear susceptibility in the flat $\delta_{1}-\delta_{4}$ of (**a**) with Re⁡(δ2)=a−. The coincidence counting rate of quadphoton with single circularly polarized dressing field. (**f**) A sectional view of four-dimensional coincidence counting rate of quadphoton. (**g**) The coincidence counting rate of (**f**) in the $\tau_{1}$ and $\tau_{2}$ directions. (**h**) The same as (**g**), but in the $\tau_{1}$ and $\tau_{3}$ directions. (**i**) The same as (**g**), but in the $\tau_{2}$ and $\tau_{3}$ directions.

**Figure 4:**
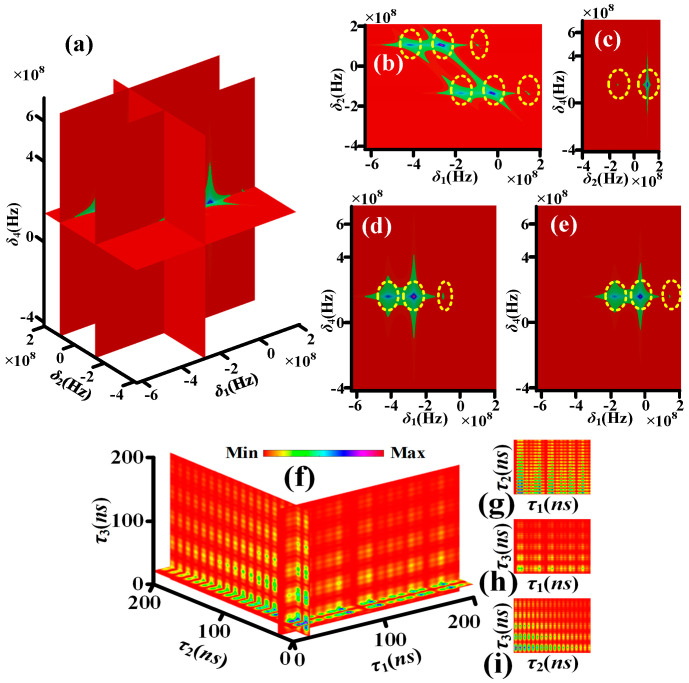
Similar to Figure 3, but (**a**) $\left|\chi_{S4DM}^{\left(7\right)}\right|$ with double circularly polarized dressing fields and (**c**) with Re⁡(δ1)=b−−a+. The coincidence counting rate of quadphoton. Similar to Figure 3, but with double circularly polarized dressing fields.

**Figure 5:**
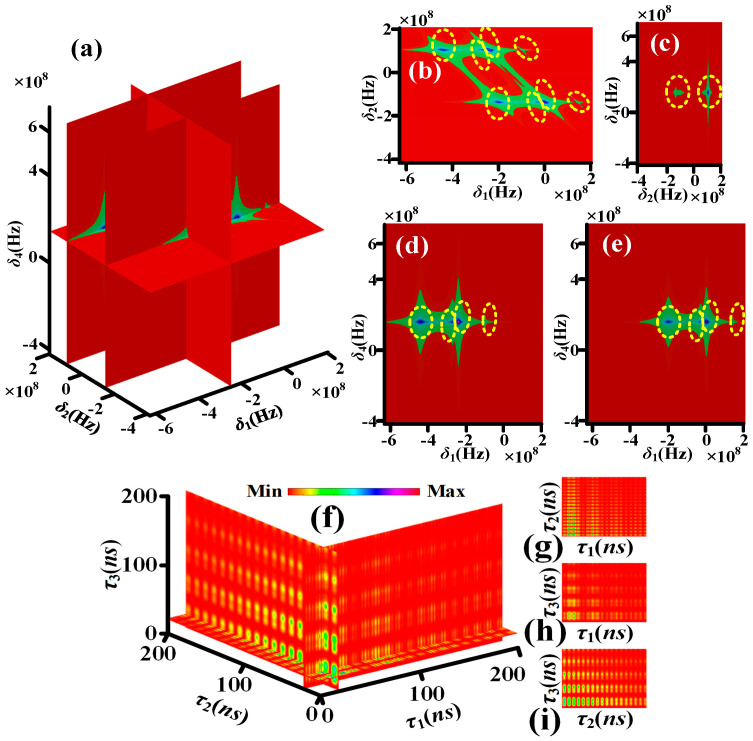
Similar to Figure 3, but (**a**) $\left|\chi_{S4TM}^{\left(7\right)}\right|$ with triple circularly polarized dressing fields and (**c**) with Re⁡(δ1)=c+−a+. The coincidence counting rate of quadphoton. Similar to Figure 3, but with triple circularly polarized dressing fields.

Figure 3a–e, simulated by Equation (S6), show the theoretical nonlinear susceptibility of quadphoton versus *δ*_1_, *δ*_2_ and *δ*_4_ controlled by single circularly polarized dressing field. Each yellow dotted circle contains one corresponding frequency mode. The four yellow dotted circles in *δ*_1_ direction of Figure 3b, two yellow dotted circles in *δ*_1_ direction of Figure 3d and two yellow dotted circles in *δ*_1_ direction of Figure 3e indicate S1 photon have four frequency modes. The two yellow dotted circles in *δ*_2_ direction of Figure 3b or Figure 3c indicate S2 photon have two frequency modes. The one yellow dotted circles in *δ*_4_ direction of Figure 3c, Figure 3d or Figure 3e indicates S4 photon has one frequency mode. It has been established that the S3 photon exhibits two distinct frequency modes in accordance with the energy conservation condition, *δ*_1_ + *δ*_2_ + *δ*_3_ + *δ*_4_ = 0. As illustrated in Figure 4a–e, the theoretical nonlinear susceptibility of the quadphoton is demonstrated versus *δ*_1_, *δ*_2_ and *δ*_4_, which are controlled by double circularly polarized dressing fields. This theoretical investigation was simulated using Equation (S7). The six yellow dotted circles in *δ*_1_ direction of Figure 4b, three yellow dotted circles in *δ*_1_ direction of Figure 4d and three yellow dotted circles in *δ*_1_ direction of Figure 4e indicate S1 photon have six frequency modes. The two yellow dotted circles in *δ*_2_ direction of Figure 4b or Figure 4c indicate S2 photon have two frequency modes. The one yellow dotted circles in *δ*_4_ direction of Figure 4c, Figure 4d or Figure 4e indicates S4 photon has one frequency mode. S3 photon has three frequency modes as per the energy conservation. Figure 5a–e, simulated by Equation (S8), show the theoretical nonlinear susceptibility of quadphoton versus *δ*_1_, *δ*_2_ and *δ*_4_ controlled by three circularly polarized dressing fields. The eight yellow dotted circles in *δ*_1_ direction of Figure 5b, four yellow dotted circles in *δ*_1_ direction of Figure 5d and four yellow dotted circles in *δ*_1_ direction of Figure 5e indicate S1 photon have eight frequency modes. The two yellow dotted circles in *δ*_2_ direction of Figure 5b or Figure 5c indicate S2 photon have two frequency modes. The one yellow dotted circles in *δ*_4_ direction of Figure 5c, Figure 5d or Figure 5e indicates S4 photon has one frequency mode. S3 photon has four frequency modes as per the energy conservation.

For modelling and demonstrating *Rcc*, polarization-based energy-time entanglement state with single, double and triple dressing can be modelled as Equations (S9)–(S11), respectively.

Based on the resonant position and line width of *δ*_i_ obtained from Table S1, the residue theorem is applied to integrate $\chi^{\left(7\right)}$ to obtain the quadphoton amplitude with single dressing (***E***_1_), double dressing (***E***_1_ and ***E***_4_) and triple dressing (***E***_1_, ***E***_4_ and ***E***_3_) as Equations(S12)–(S14), respectively. Where *τ*_1_ = *t_S_*_1_ − *t_S_*_4_, *τ*_2_ = *t_S_*_2_ − *t_S_*_4_ and *τ*_3_ = *t_S_*_3_ − *t_S_*_4_. Afterwards, *Rcc* for quadphoton with single, double and triple dressing can be separately expressed as follows.

(13) $${Rcc}_{SM}\left(\tau_{1},\tau_{2},\tau_{3}\right)=\underset{T\to \infty }{\lim}\frac{1}{T}\int_{0}^{T}d\tau_{1}\int_{0}^{T}d\tau_{2}\int_{0}^{T}d\tau_{3}{\left|B_{SM}\left(\tau_{1},\tau_{2},\tau_{3}\right)\right|}^{2}$$

(14) $${Rcc}_{DM}\left(\tau_{1},\tau_{2},\tau_{3}\right)=\underset{T\to \infty }{\lim}\frac{1}{T}\int_{0}^{T}d\tau_{1}\int_{0}^{T}d\tau_{2}\int_{0}^{T}d\tau_{3}{\left|B_{DM}\left(\tau_{1},\tau_{2},\tau_{3}\right)\right|}^{2}$$

(15) $${Rcc}_{TM}\left(\tau_{1},\tau_{2},\tau_{3}\right)=\underset{T\to \infty }{\lim}\frac{1}{T}\int_{0}^{T}d\tau_{1}\int_{0}^{T}d\tau_{2}\int_{0}^{T}d\tau_{3}{\left|B_{TM}\left(\tau_{1},\tau_{2},\tau_{3}\right)\right|}^{2}$$

For characterizing the polarization-based energy-time-entangled quadphoton, we thus simulate *Rcc* according to Equations (13)–(15). Figure 3f shows the four-dimensional (4D) simulation of coincidence counting rate with single circularly polarized dressing field (Equation (13)). The wave form displays multiple decaying Rabi oscillations with four periods of $2\pi /\Omega_{e1M}$, $2\pi /\Delta_{3}$, $2\pi /\left|\Delta_{3}-\Omega_{e1M}\right|$ and $2\pi /(\Delta_{3}+\Omega_{e1M})$ in *τ*_1_ direction of Figure 3f, Figure 3g or Figure 3h.

The Rabi oscillation, comprising four periods, is induced by the energy exchange and destructive interference among four polarization-based frequency modes of a single photon from four polarization-based coherent channels of energy conservation (*C*_1_–*C*_4_), as illustrated in Figure 2a. The wave form in *τ*_2_ (*τ*_3_) direction of Figure 3f, Figure 3g or Figure 3i (Figure 3f, Figure 3h or Figure 3i presents decaying Rabi oscillation with single period of $2\pi /\Omega_{e1M}$ ($2\pi /\Delta_{3}$). This phenomenon is attributable to the energy exchange and destructive interference between two polarization-based frequency modes of S2 (S3) photons from *C*_1_–*C*_4_, as illustrated in Figure 2a. Figure 4f presents the 4D simulation of coincidence counting rate with double circularly polarized dressing fields (Equation (14)). The waveform displays multiple decaying Rabi oscillations with ten periods in the τ1 direction of Figure 4f, Figure 4g or Figure 4h. The ten periods are $2\pi /\Omega_{e1M}$, $2\pi /\Omega_{e2M}$, $2\pi /\left|\Omega_{e2M}-\Omega_{e1M}\right|$, $4\pi /(\Delta_{4}+2\Delta_{3}+\Omega_{e2M})$, $4\pi /\left|\Delta_{4}+2\Delta_{3}+\Omega_{e2M}-2\Omega_{e1M}\right|$, $2\pi /(\Omega_{e2M}+\Omega_{e1M})$, $4\pi /(\Delta_{4}+2\Delta_{3}+\Omega_{e2M}+2\Omega_{e1M})$, $4\pi /\left|\Delta_{4}+2\Delta_{3}-\Omega_{e2M}\right|$, $4\pi /\left|\Delta_{4}+2\Delta_{3}-\Omega_{e2M}-2\Omega_{e1M}\right|$ and $4\pi /\left|\Delta_{4}+2\Delta_{3}-\Omega_{e2M}+2\Omega_{e1M}\right|$. The Rabi oscillations with ten periods are induced via the energy exchange and destructive interference among six polarization-based frequency modes of S1 photon from six polarization-based coherent channels of energy conservation (*C*_5_–*C*_10_) schemed in Figure 2b. The wave form in *τ*_2_ direction of Figure 4f, Figure 4g or Figure 4i presents decaying Rabi oscillation with single period of $2\pi /\Omega_{e1M}$. The wave form in *τ*_3_ direction of Figure 4f, Figure 4h or Figure 4i presents decaying Rabi oscillations with three periods of $2\pi /\Omega_{e2M}$, $4\pi /(\Delta_{4}+2\Delta_{3}+\Omega_{e2M})$ and $4\pi /\left|\Delta_{4}+2\Delta_{3}-\Omega_{e2M}\right|$. The phenomenon is for the reason of the energy exchange and destructive interference between three polarization-based frequency modes of S3 photon from *C*_5_–*C*_10_ schemed in Figure 2b. Figure 5f reveals the 4D simulation of coincidence counting rate with triple circularly polarized dressing fields (Equation (15)). The wave form displays multiple decaying Rabi oscillations with nineteen periods in *τ*_1_ direction of Figure 5f, Figure 4g or Figure 4h. The nineteen periods are $2\pi /\Omega_{e1M}$, $2\pi /\Omega_{e2M}$, $2\pi /\left|\Omega_{e2M}-\Omega_{e1M}\right|$, $4\pi /\left|\Delta_{3}+\Delta_{4}-\Omega_{e3M}+\Omega_{e2M}\right|$, $4\pi /\left|\Delta_{3}+\Delta_{4}-\Omega_{e3M}+\Omega_{e2M}-2\Omega_{e1M}\right|$, $4\pi /(\Delta_{3}+\Delta_{4}+\Omega_{e3M}+\Omega_{e2M})$, $4\pi /\left|\Delta_{3}+\Delta_{4}+\Omega_{e3M}+\Omega_{e2M}-2\Omega_{e1M}\right|$, $2\pi /(\Omega_{e2M}+\Omega_{e1M})$, $4\pi /\left|\Delta_{3}+\Delta_{4}-\Omega_{e3M}+\Omega_{e2M}+2\Omega_{e1M}\right|$, $4\pi /(\Delta_{3}+\Delta_{4}+\Omega_{e3M}+\Omega_{e2M}+2\Omega_{e1M})$, $4\pi /\left|\Delta_{3}+\Delta_{4}-\Omega_{e3M}-\Omega_{e2M}\right|$, $4\pi /\left|\Delta_{3}+\Delta_{4}-\Omega_{e3M}-\Omega_{e2M}-2\Omega_{e1M}\right|$, $4\pi /\left|\Delta_{3}+\Delta_{4}+\Omega_{e3M}-\Omega_{e2M}\right|$, $4\pi /\left|\Delta_{3}+\Delta_{4}+\Omega_{e3M}-\Omega_{e2M}-2\Omega_{e1M}\right|$, $4\pi /\left|\Delta_{3}+\Delta_{4}-\Omega_{e3M}-\Omega_{e2M}+2\Omega_{e1M}\right|$, $4\pi /\left|\Delta_{3}+\Delta_{4}+\Omega_{e3M}-\Omega_{e2M}+2\Omega_{e1M}\right|$, $2\pi /\Omega_{e3M}$, $2\pi /\left|\Omega_{e3M}-\Omega_{e1M}\right|$, and $2\pi /(\Omega_{e3M}+\Omega_{e1M})$. The Rabi oscillations, which exhibit nineteen periods, are induced through the energy exchange and destructive interference among eight polarization-based frequency modes of S1 photons from eight polarization-based coherent channels of energy conservation (*C*_11_–*C*_18_), as illustrated in Figure 2c. The wave form in *τ*_2_ direction of Figure 5f, Figure 5g or Figure 5i presents decaying Rabi oscillation with single period of $2\pi /\Omega_{e1M}$. The wave form in *τ*_3_ direction of Figure 5f, Figure 5h or Figure 5i presents decaying Rabi oscillations with six periods of $2\pi /\Omega_{e2M}$, $4\pi /\left|\Delta_{3}+\Delta_{4}-\Omega_{e3M}+\Omega_{e2M}\right|$, $4\pi /(\Delta_{3}+\Delta_{4}+\Omega_{e3M}+\Omega_{e2M})$, $4\pi /\left|\Delta_{3}+\Delta_{4}-\Omega_{e3M}-\Omega_{e2M}\right|$, $4\pi /\left|\Delta_{3}+\Delta_{4}+\Omega_{e3M}-\Omega_{e2M}\right|$ and $2\pi /\Omega_{e3M}$. The phenomenon under investigation is attributed to the energy exchange and destructive interference between four polarization-based frequency modes of S3 photons from *C*_11_–*C*_18_, as depicted in Figure 2c. Indeed, distinguishing between these periods can be challenging. For instance, there are ten periods of S1 photon with double dressing fields, nineteen periods of S1 photon, and six periods of S3 photon with triple dressing fields. This difficulty arises from the presence of certain periods that are either too small or too large, several periods that are quite close together, and small periods that are covered by large periods.

### 2.5. The Generation of Polarization-Based High-Dimensional Entanglement Through Passive Non-Hermitian Processes

The EIT can not only promote the EWM process but also structure a passive non-Hermitian system. The polarized dressing terms $\Gamma_{41M}+i\delta_{2}+i\Delta_{1}+(\frac{\sqrt{5}}{6}G_{1M}{)}^{2}(2\mathrm{co}s^{2}\theta \sin^{2}\theta )/(\Gamma_{21M}+i\delta_{2})$, $\Gamma_{41M}+i\delta_{2}+i\delta_{1}+i\Delta_{4}+(\frac{2}{3}\sqrt{\frac{1}{5}}G_{4M}{)}^{2}(2\mathrm{co}s^{2}\theta \sin^{2}\theta )/(\Gamma_{11M}+i\delta_{2}+i\delta_{1})$ and $\Gamma_{51M}+i\delta_{2}+i\delta_{1}+i\Delta_{4}+i\Delta_{3}+(\frac{8}{15}\sqrt{\frac{2}{21}}G_{3M}{)}^{2}(2\mathrm{co}s^{2}\theta \sin^{2}\theta )/(\Gamma_{41M}+i\delta_{2}+i\delta_{1}+i\Delta_{4})$ of susceptibility in Equation (S8) are equivalent to EIT-based passive non-Hermitian system. By minimizing triple polarized dressing terms, the eigenvalues can be derived as Equations (S15) and (S16).

In Figures S1 and S2, the evolution of the real and imaginary parts of the eigenvalues is demonstrated in the parameter space [*G*_1*θM*_, *G*_4*θM*_, *G*_3*θM*_]. *δ*_1i_ represents the *i*th eigenvalue of *δ*_1_. When [*G*_1*θM*_, *G*_4*θM*_, *G*_3*θM*_] = [$\left|g_{1\theta M}\right|$, $\left|g_{4\theta M}\right|$, $\left|g_{3\theta M}\right|$] = [0.5Γ_21*M*_, 0.5Γ_11*M*_, 0.38Γ_41*M*_] is satisfied, triple non-Hermitian EP_1_ (EP_11_, EP_12_), EP_2_ (EP_21_, EP_22_) and EP_3_ (EP_31_, EP_32_) emerge, being followed by the degeneracy of three pairs of eigenvalues. Where *g*_1*θM*_ = Re⁡[[−[iΓ21M+Γ41M−Δ1]2/4−(Γ41M+iΔ1)]1/2], *g*_4*θM*_ = Re⁡[[−[iΓ11M+Γ41M−Δ4]2/4−(Γ41M+iΔ4)]1/2], *g*_3*θM*_ = Re⁡[[−[iΓ41M+Γ51M−2Δ4−Δ3]2/4−[Γ51M+i(Δ4+Δ3+Γ51MΔ4)−Δ42−Δ4Δ3]]1/2]. Thus, it can obtain three second-order non-Hermitian EPs. It has been demonstrated that, under certain conditions, the real parts of the eigenvalues undergo a process of splitting. These conditions include the satisfaction of the following inequalities: [*G*_1*θM*_, *G*_4*θM*_, *G*_3*θM*_] > [0.5Γ_21*M*_, 0.5Γ_11*M*_, 0.38Γ_41*M*_], as demonstrated in *G*_1*θM*_/Γ_21*M*_ direction of the combination of Figures S1b,d (Figures S1j,l and S2b,d,j,l), *G*_4*θM*_/Γ_11*M*_ direction of the combination of Figures S1b,j and S1d,l, as well as *G*_3*θM*_/Γ_41*M*_ direction of the combination of Figures S2b,j and S2d,l; while the imaginary parts remain degenerate, as demonstrated in *G*_1*θM*_/Γ_21*M*_ direction of the combination of Figure S1f,h (Figures S1n,p and S2f,h,m,p), *G*_4*θM*_/Γ_11*M*_ direction of the combination of Figure S1f,n,h,p as well as *G*_3*θM*_/Γ_41*M*_ direction of the combination of Figure S2f,n,h,p. The system exhibits three second-order non-Hermitian quasi parity–time (PT) symmetries. In the event of encountering the following relation, the imaginary parts of the eigenvalues undergo splitting: [*G*_1*θM*_, *G*_4*θM*_, *G*_3*θM*_] < [0.5Γ_21*M*_, 0.5Γ_11*M*_, 0.38Γ_41*M*_], as demonstrated in *G*_1*θM*_/Γ_21*M*_ direction of the combination of Figure S1f,h (Figures S1n,p and S2f,h,n,p) *G*_4*θM*_/Γ_11*M*_ direction of the combination of Figure S1f,n,h,p as well as *G*_3*θM*_/Γ_41*M*_ direction of the combination of Figure S2f,n,h,p; while the real parts keep degenerate, as demonstrated in *G*_1*θM*_/Γ_21*M*_ direction of the combination of Figure S1b,d (Figures S1j,l and S2b,d,j,l), *G*_4*θM*_/Γ_11*M*_ direction of the combination of Figure S1b,j,d,l as well as *G*_3*θM*_/Γ_41*M*_ direction of the combination of Figure S2b,j,d,l. The outcome of this process is the breakdown of three second-order non-Hermitian quasi-PT symmetries within the system. In summary, the evolution of three second-order non-Hermitian EPs can be controlled by regulating the triple-polarized dressing fields, thereby determining whether the breakdown of three second-order non-Hermitian quasi-PT symmetries occurs. Considering Equation (S8), *δ*_2_ and *δ*_4_ have two eigenvalues and one eigenvalue, respectively. The eigenvalues of *δ*_3_ can be thus obtained via the polarization-based energy conservation.

**Figure 6:**
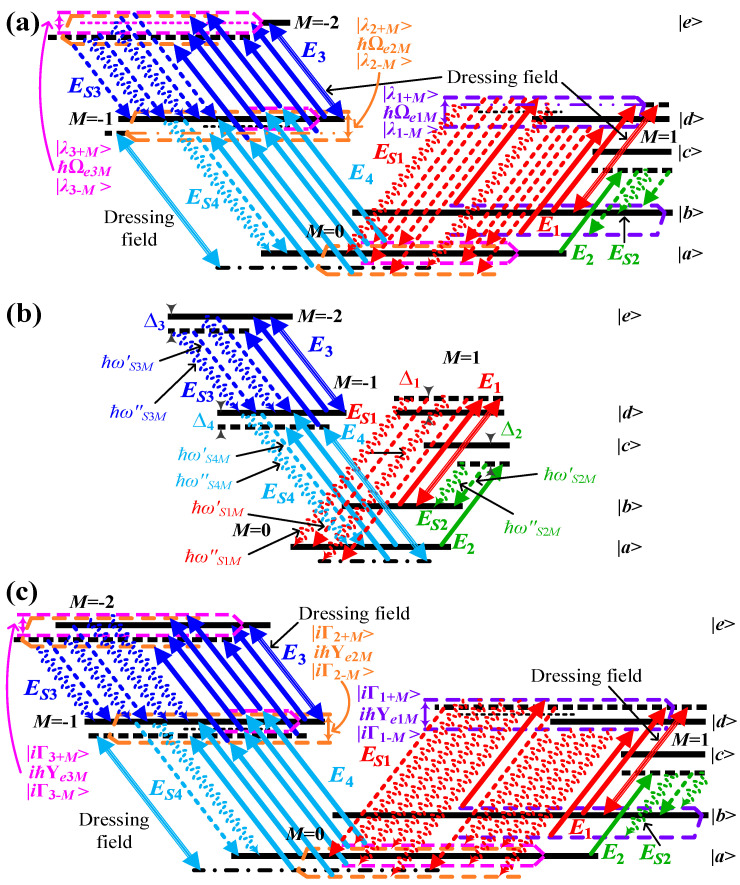
Energy-level sketch of multi-resonance (real part) and multi-absorptive (imaginary part) coherent channels in the EWM process is presented herein. (**a**) Eight-resonance and two-absorptive channels at [*G*_1*θM*_, *G*_4*θM*_, *G*_3*θM*_] > [0.5Γ_21*M*_, 0.5Γ_11*M*_, 0.38Γ_41*M*_]. (**b**) two-resonance and two-absorptive channels at [*G*_1*θM*_, *G*_4*θM*_, *G*_3*θM*_] = [0.5Γ_21*M*_, 0.5Γ_11*M*_, 0.38Γ_41*M*_]. (**c**) Two-resonance and eight-absorptive channels at [*G*_1*θM*_, *G*_4*θM*_, *G*_3*θM*_] < [0.5Γ_21*M*_, 0.5Γ_11*M*_, 0.38Γ_41*M*_].

According to the above theory concerning the passive non-Hermitian system, in atomic polarized energy levels, we naturally structure multi-resonance and multi-absorptive coherent channels of quadphotons. As illustrated in Figure 6, the rubidium atomic polarized energy-level diagrams of multi-resonance and multi-absorptive coherent channels of quadphoton are depicted in the “dressed-state” picture with triple circularly polarized dressing fields. With [*G*_1*θM*_, *G*_4*θM*_, *G*_3*θM*_] > [0.5Γ_21*M*_, 0.5Γ_11*M*_, 0.38Γ_41*M*_], the real components of the energy levels |*d*, *M* = 1⟩, |*d*, *M* = −1⟩ and |*e*, *M* = –2⟩ are split into energy levels |*λ*_1±*M*_⟩, |*λ*_2±*M*_⟩ and |*λ*_3±*M*_⟩, as shown in Figure 6a. It is noteworthy that the corresponding imaginary components remain degenerate. Thus, S1 photon has eight resonance dressed states ($\hbar ({\varpi^{\prime }}_{S1M}+b_{\pm }-a_{\pm }+i\Gamma_{e5\pm M}-i\Gamma_{e4\pm M})$, $\hbar ({\varpi^{\prime }}_{S1M}+c_{\pm }-a_{\pm }+i\Gamma_{e6\pm M}-i\Gamma_{e4\pm M})$) and two absorptive states ($\hbar ({\varpi^{\prime \prime }}_{S1M}+\Gamma_{e2M}-\Gamma_{e1M}+i(\Delta_{1}-\Delta_{4})/2)$, $\hbar ({\varpi^{\prime \prime }}_{S1M}+\Gamma_{e3M}-\Gamma_{e1M}+i(\Delta_{1}-\Delta_{3}-2\Delta_{4})/2)$), S2 photon has two resonance dressed states $\hbar ({\varpi^{\prime }}_{S2M}+a_{\pm }+i\Gamma_{e4\pm M})$ and one absorptive state $\hbar ({\varpi^{\prime \prime }}_{S2M}+\Gamma_{e1M}-i\Delta_{1}/2)$, S3 photon has four resonance dressed states ($\hbar ({\varpi^{\prime }}_{S3M}-b_{\pm }-\Delta_{4}-i\Gamma_{e5\pm M}+i\Gamma_{41M})$, $\hbar ({\varpi^{\prime }}_{S3M}-c_{\pm }-\Delta_{4}-i\Gamma_{e6\pm M}+i\Gamma_{41M})$) and two absorptive states ($\hbar ({\varpi^{\prime \prime }}_{S3M}+\Gamma_{41M}-\Gamma_{e2M}-i\Delta_{4}/2)$, $\hbar ({\varpi^{\prime \prime }}_{S3M}+\Gamma_{41M}-\Gamma_{e3M}+i\Delta_{3}/2)$), and S4 photon has one resonance state $\hbar ({\varpi^{\prime }}_{S4M}+\Delta_{4}-i\Gamma_{41M})$ and one absorptive state $\hbar ({\varpi^{\prime \prime }}_{S4M}-\Gamma_{41M}+i\Delta_{4})$. All output and compatible input states form eight polarization-based resonance coherent channels and two polarization-based absorptive coherent channels. When [*G*_1*θM*_, *G*_4*θM*_, *G*_3*θM*_] = [0.5Γ_21*M*_, 0.5Γ_11*M*_, 0.38Γ_41*M*_], the real and imaginary components of all energy levels remain degenerate, as demonstrated in Figure 6b. Consequently, the S1 photon exhibits two resonance states ($\hbar ({\varpi^{\prime }}_{S1;EP_{41}M}+(\Delta_{1}-\Delta_{4})/2+i\Gamma_{e2M}-i\Gamma_{e1M})$, $\hbar ({\varpi^{\prime }}_{S1;EP_{51}M}+(\Delta_{1}-\Delta_{3}-2\Delta_{4})/2+i\Gamma_{e3M}-i\Gamma_{e1M})$), and two absorptive states ($\hbar ({\varpi^{\prime \prime }}_{S1;EP_{42}M}+\Gamma_{e2M}-\Gamma_{e1M}+i(\Delta_{1}-\Delta_{4})/2)$, $\hbar ({\varpi^{\prime \prime }}_{S1;EP_{52}M}+\Gamma_{e3M}-\Gamma_{e1M}+i(\Delta_{1}-\Delta_{3}-2\Delta_{4})/2)$). The S2 photon has one additional resonance state, given by $\hbar ({\varpi^{\prime }}_{S2;EP_{i1}M}-\Delta_{1}/2+i\Gamma_{e1M})$ and one absorptive state $\hbar ({\varpi^{\prime \prime }}_{S2;EP_{i2}M}+\Gamma_{e1M}-i\Delta_{1}/2)$. S3 photon has two resonance states ($\hbar ({\varpi^{\prime }}_{S3;EP_{41}M}-\Delta_{4}/2+i\Gamma_{41M}-i\Gamma_{e2M})$, $\hbar ({\varpi^{\prime }}_{S3;EP_{51}M}+\Delta_{3}/2+i\Gamma_{41M}-i\Gamma_{e3M})$) and two absorptive states ($\hbar ({\varpi^{\prime \prime }}_{S3;EP_{42}M}+\Gamma_{41M}-\Gamma_{e2M}-i\Delta_{4}/2)$, $\hbar ({\varpi^{\prime \prime }}_{S3;EP_{52}M}+\Gamma_{41M}-\Gamma_{e3M}+i\Delta_{3}/2)$), and S4 photon has one resonance state $\hbar ({\varpi^{\prime }}_{S4;EP_{i1}M}+\Delta_{4}-i\Gamma_{41M})$ and one absorptive state $\hbar ({\varpi^{\prime \prime }}_{S4;EP_{i2}M}-\Gamma_{41M}+i\Delta_{4})$. All output and matched input states form two polarization-based resonance coherent channels and two polarization-based absorptive coherent channels. It is evident that with [*G*_1*θM*_, *G*_4*θM*_, *G*_3*θM*_] < [0.5Γ_21*M*_, 0.5Γ_11*M*_, 0.38Γ_41*M*_], the imaginary components of the energy levels |*d*, *M* = 1⟩, |*d*, *M* = −1⟩ and |*e*, *M* = −2⟩ are split into energy levels |Г_1 ± *M*_⟩, |Г_2 ± *M*_⟩ and |Г_3 ± *M*_⟩ as illustrated in Figure 6c. It is important to note that the corresponding real components remain degenerate. Accordingly, S1 photon has eight absorptive dressed states ($\hbar ({\varpi^{\prime \prime }}_{S1M}+e_{\pm }-d_{\pm }+i\Delta_{e2\pm }-i\Delta_{e1\pm })$, $\hbar ({\varpi^{\prime \prime }}_{S1M}+f_{\pm }-d_{\pm }+i\Delta_{e3\pm }-i\Delta_{e1\pm })$) and two resonance states ($\hbar ({\varpi^{\prime }}_{S1M}+(\Delta_{1}-\Delta_{4})/2+i\Gamma_{e2M}-i\Gamma_{e1M})$, $\hbar ({\varpi^{\prime }}_{S1M}+(\Delta_{1}-\Delta_{3}-2\Delta_{4})/2+i\Gamma_{e3M}-i\Gamma_{e1M})$), S2 photon has two absorptive dressed states $\hbar ({\varpi^{\prime \prime }}_{S2M}+d_{\pm }+i\Delta_{e1\pm })$ and one resonance state $\hbar ({\varpi^{\prime }}_{S2M}-\Delta_{1}/2+i\Gamma_{e1M})$, S3 photon has four absorptive dressed states ($\hbar ({\varpi^{\prime \prime }}_{S3M}+\Gamma_{41M}-e_{\pm }-i\Delta_{4}-i\Delta_{e2\pm })$, $\hbar ({\varpi^{\prime \prime }}_{S3M}+\Gamma_{41M}-f_{\pm }-i\Delta_{4}-i\Delta_{e3\pm })$) and two resonance states ($\hbar ({\varpi^{\prime }}_{S3M}-\Delta_{4}/2+i\Gamma_{41M}-i\Gamma_{e2M})$, $\hbar ({\varpi^{\prime }}_{S3M}+\Delta_{3}/2+i\Gamma_{41M}-i\Gamma_{e3M})$), and S4 photon has one absorptive state $\hbar ({\varpi^{\prime \prime }}_{S4M}-\Gamma_{41M}+i\Delta_{4})$ and one resonance state $\hbar ({\varpi^{\prime }}_{S4M}+\Delta_{4}-i\Gamma_{41M})$. Similarly, it can generate eight polarization-based absorptive coherent channels and two polarization-based resonance coherent channels. Where $\Gamma_{e4\pm M}=(\Gamma_{41M}+\Gamma_{21M})/2+\Delta_{1}\Gamma_{21M}/(2a_{\pm })$, $\Gamma_{e5\pm M}=(\Gamma_{41M}+\Gamma_{11M})/2+\Delta_{4}\Gamma_{11M}/(2b_{\pm })$, $\Gamma_{e6\pm M}=(\Gamma_{51M}+\Gamma_{41M})/2+[\Gamma_{41M}(\Delta_{4}+\Delta_{3})+\Gamma_{51M}\Delta_{4}]/(2c_{\pm })$, $d_{\pm }=(\Gamma_{41M}+\Gamma_{21M}\pm Y_{e1M})/2$, $\Delta_{e1\pm }=-\Delta_{1}/2+\Delta_{1}\Gamma_{21M}/(2d_{\pm })$, $e_{\pm }=(\Gamma_{41M}+\Gamma_{11M}\pm Y_{e2M})/2$, $\Delta_{e2\pm }=-\Delta_{4}/2+\Delta_{4}\Gamma_{11M}/(2e_{\pm })$, $f_{\pm }=(\Gamma_{51M}+\Gamma_{41M}\pm Y_{e3M})/2$, $\Delta_{e3\pm }=-(2\Delta_{4}+\Delta_{3})/2+[\Gamma_{41M}(\Delta_{4}+\Delta_{3})+\Gamma_{51M}\Delta_{4}]/(2f_{\pm })$, $Y_{e1M}=\sqrt{{Y_{e1M}}^{\prime }}$, $Y_{e1M}\prime =(\Gamma_{41M}+\Gamma_{21M}{)}^{2}-4(\Gamma_{41M}\Gamma_{21M}+(\frac{\sqrt{5}}{6}G_{1M}{)}^{2}(2\mathrm{co}s^{2}\theta \sin^{2}\theta ))$, $Y_{e2M}=\sqrt{{Y_{e2M}}^{\prime }}$, ${Y_{e2M}}^{\prime }=(\Gamma_{41M}+\Gamma_{11M}{)}^{2}-4(\Gamma_{41M}\Gamma_{11M}+(\frac{2}{3}\sqrt{\frac{1}{5}}G_{4M}{)}^{2}(2\mathrm{co}s^{2}\theta \sin^{2}\theta ))$, $Y_{e3M}=\sqrt{Y_{e3M}\prime }$ and ${Y_{e3M}}^{\prime }=(\Gamma_{51M}+\Gamma_{41M}{)}^{2}-4(\Gamma_{51M}\Gamma_{41M}-\Delta_{4}(\Delta_{4}+\Delta_{3})+(\frac{8}{15}\sqrt{\frac{2}{21}}G_{3M}{)}^{2}(2\mathrm{co}s^{2}\theta \sin^{2}\theta ))$. Table S2 supplies the relevant resonance and absorptive coherent channels of quadphotons in detail.

**Figure 7:**
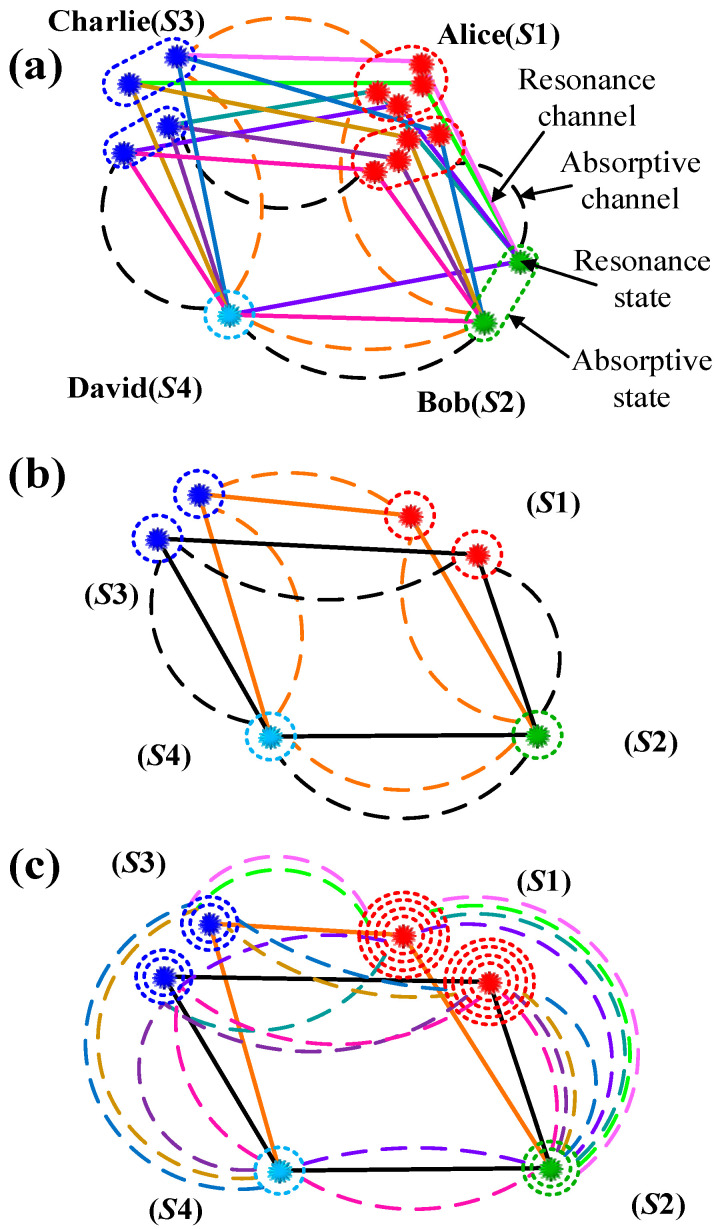
As illustrated in (**a**–**c**), the model of a polarization-based high-dimensional four-body entangled quantum network has been constructed by applying the entanglement states depicted in Figure 6a–c.

Ulteriorly, the preparative entanglement states are used to structure polarization-based high-dimensional four-body entangled quantum network element among Alice, Bob, Charlie and David. The states of S1–4 photons function as the information carriers for Alice, Bob, Charlie, and David, respectively. The states of the photons are represented by [*G*_1*θM*_, *G*_4*θM*_, *G*_3*θM*_] > [0.5Γ_21*M*_, 0.5Γ_11*M*_, 0.38Γ_41*M*_], as illustrated in Figure 7a, eight resonance dressed states of the S1 photon, two resonance dressed states of the S2 photon, four resonance dressed states of the S3 photon, and one resonance state of the S4 photon form an eight-resonance-channels-based eight-dimensional four-body entangled communication, with an information capacity of ~65536. Two absorptive states of the S1 photon, one absorptive state of the S2 photon, two absorptive states of the S3 photon, and one absorptive state of the S4 photon form a two-absorptive-channels-based two-dimensional four-body entangled communication, with an information capacity of ~16. With [*G*_1*θM*_, *G*_4*θM*_, *G*_3*θM*_] = [0.5Γ_21*M*_, 0.5Γ_11*M*_, 0.38Γ_41*M*_], as illustrated in Figure 7b, two resonance (absorptive) states of S1 photon, one resonance (absorptive) state of S2 photon, two resonance (absorptive) states of S3 photon, and one resonance (absorptive) state of S4 photon comprise two-resonance(absorptive)-channels-based two-dimensional four-body entangled communication, its information capacity is ~16. As illustrated in Figure 7c, the information capacity of this communication is maximized when the following conditions are met: [*G*_1*θM*_, *G*_4*θM*_, *G*_3*θM*_] < [0.5Γ_21*M*_, 0.5Γ_11*M*_, 0.38Γ_41*M*_] in which the eight absorptive dressed states of the S1 photon, the two absorptive dressed states of the S2 photon, the four absorptive dressed states of the S3 photon, and the one absorptive state of the S4 photon collectively constitute the eight-absorptive-channels-based eight-dimensional four-body entangled communication, with an information capacity of ~65,536. Analogously, this configuration can also facilitate two-resonance-channels-based two-dimensional four-body entangled communication, with an information capacity of ~16.

## 3. Conclusions

In summary, we theoretically demonstrated EWM-based hyper-entangled quadphotons generation in ^85^Rb vapor. Polarization-based energy-time entanglement was obtained under single, double and triple circularly polarized dressing fields. We quantized the degenerate Zeeman sub states for quadphotons along with polarization-based energy-time entanglement, which formed multiple coherent channels. These coherent channels were regulated through dressing and assisted by polarized nonlinear susceptibilities while satisfying polarization-based energy conservation. Ulteriorly, polarization-based tunable high-dimensional four-body entanglement and quantum network elements were demonstrated through passive non-Hermitian characteristic by simulating multi-resonance (real part) and multi-absorptive (imaginary part) coherent channels under triple dressing regulation. The fine physical characteristics make the hyper-entangled quadphoton naturally serve as a significant entangled source. The predicted high information capacity is stimulated by coexistent hyper and high-dimensional entanglements of genuine quadphoton. The high producing efficiency is caused by four strong input fields, EIT slow light effect, and etc. The atomic passive non-Hermitian characteristic eventuates the system with a high tunability around EPs under triple dressing regulation. Eventually, the produced hyper-entangled quadphoton is potential to be applied in tunable four-body entangled quantum networks with high information capacity, whose multi-partite entanglement and multiple-degree-of-freedom properties help optimize the accuracy of quantum sensors.

## Data Availability

Data underlying the results presented in this paper are not publicly available at this time but may be obtained from the authors upon reasonable request.

## Supplementary Materials

The following supporting information can be downloaded at: https://www.mdpi.com/article/10.3390/s25113425/s1.
