# Supplementary material for: Direct Generation and Non-Hermitian Regulation of Energy-Time-Polarization-Hyper-Entangled Quadphotons
Source: Sensors (Basel). 2025 May 29;25(11):3425. doi: 10.3390/s25113425 (PMC12158328; doi:10.3390/s25113425)
Supplement: Supplementary file 1 [file sensors-25-03425-s001.zip › sensors-3568626-supplementary.pdf]

Direct generation and non-Hermitian regulation of energy-time-polarization-hyper-entangled quadphotons: supplemental document

The conditioned three-photon (with one of quadphoton undetected) correlation plays a prominent role in the quantum characteristics of the quadphoton state. The three-photon coincidence counting rates in quadphoton state can be separately expressed as

$$\begin{aligned} &Rcc_{3M}(\tau_1,\tau_2) \\ &= \lim_{T\to\infty}\frac{1}{T}\int_0^T d\tau_1\int_0^T d\tau_2 \sum_{\mathbf{k}_{S3}} \left|\langle 0| E_{S4}^{(+)}\varsigma(\mathbf{k}_{S3})E_{S2}^{(+)}E_{S1}^{(+)}|\psi\rangle\right|^2 \\ &= R_{30}\int d\delta_3 \left|\int d\delta_1 \int d\delta_2 \kappa(\delta_1,\delta_2,\delta_3)\Phi(\delta_1,\delta_2,\delta_3)e^{-i(\delta_1\tau_1+\delta_2\tau_2)}\right|^2 \end{aligned} \quad \textbf{(S1)}$$

$$\begin{aligned} &Rcc_{3M}(\tau_1,\tau_3) \\ &= \lim_{T\to\infty}\frac{1}{T}\int_0^T d\tau_1\int_0^T d\tau_3 \sum_{\mathbf{k}_{S2}} \left|\langle 0| E_{S4}^{(+)}E_{S3}^{(+)}\varsigma(\mathbf{k}_{S2})E_{S1}^{(+)}|\psi\rangle\right|^2 \\ &= R_{30}\int d\delta_2 \left|\int d\delta_1 \int d\delta_3 \kappa(\delta_1,\delta_2,\delta_3)\Phi(\delta_1,\delta_2,\delta_3)e^{-i(\delta_1\tau_1+\delta_3\tau_3)}\right|^2 \end{aligned} \quad \textbf{(S2)}$$

$$\begin{aligned} &Rcc_{3M}(\tau_2,\tau_3) \\ &= \lim_{T\to\infty}\frac{1}{T}\int_0^T d\tau_2\int_0^T d\tau_3 \sum_{\mathbf{k}_{S1}} \left|\langle 0| E_{S4}^{(+)}E_{S3}^{(+)}E_{S2}^{(+)}\varsigma(\mathbf{k}_{S1})|\psi\rangle\right|^2 \\ &= R_{30}\int d\delta_1 \left|\int d\delta_2 \int d\delta_3 \kappa(\delta_1,\delta_2,\delta_3)\Phi(\delta_1,\delta_2,\delta_3)e^{-i(\delta_2\tau_2+\delta_3\tau_3)}\right|^2 \end{aligned} \quad \textbf{(S3)}$$

where the summation of wavenumber will be converted into integral of angular frequency $\sum_{k_{S\mathrm{i}}} \to V^{1/3}\int d\omega_{S\mathrm{i}}/(2\pi\upsilon_{S\mathrm{i}})$; $\upsilon_{S\mathrm{i}}$ is the speed of Si photon. R_{30} is a constant coefficient. The coincidence counting rates of three-photons (with one of quadphoton undetected) in quadphoton state share the same shapes of functions with those of triphotons. Such characteristics indicate that the quadphoton state generated from EWM, not only is entangled in polarization-based energy but also can degenerate to tripartite states with one of quadphoton undetected, and the quadphoton state presents an averaged distribution of tripartite states.

$$\begin{aligned} &\rho_{a_M a_M} \xrightarrow{-\frac{1}{3}G_{ac}^{+1}} \rho_{c_{M+1}a_M} \xrightarrow{\frac{1}{3}G_{cb}^{-1}} \rho_{b_M a_M} \xrightarrow{\frac{\sqrt{5}}{6}G_{bd}^{+1}} \rho_{d_{M+1}a_M} \xrightarrow{\frac{2}{3}\sqrt{\frac{1}{5}}G_{da}^{-1}} \rho_{a_M a_M} \\ &\xrightarrow{\frac{2}{3}\sqrt{\frac{1}{5}}G_{ad}^{-1}} \rho_{d_{M-1}a_M} \xrightarrow{\frac{8}{15}\sqrt{\frac{2}{21}}G_{de}^{-1}} \rho_{e_{M-2}a_M} \xrightarrow{\frac{8}{15}\sqrt{\frac{2}{21}}G_{ed}^{+1}} \rho_{d_{M-1}a_M}\ (M=-2,-1,0,1) \end{aligned} \quad \textbf{(S4)}$$

$$\begin{aligned} &\rho_{a_M a_M} \xrightarrow{(-\frac{1}{3}G_{ac}^{+1})^*} \rho_{a_M c_{M+1}} \xrightarrow{\frac{1}{3}G_{cb}^{-1}} \rho_{a_M b_M} \xrightarrow{(\frac{\sqrt{5}}{6}G_{bd}^{+1})^*} \rho_{a_M d_{M+1}} \xrightarrow{\frac{2}{3}\sqrt{\frac{1}{5}}G_{da}^{-1}} \rho_{a_M a_M} \\ &\xrightarrow{(\frac{2}{3}\sqrt{\frac{1}{5}}G_{ad}^{-1})^*} \rho_{a_M d_{M-1}} \xrightarrow{(\frac{8}{15}\sqrt{\frac{2}{21}}G_{de}^{-1})^*} \rho_{a_M e_{M-2}} \xrightarrow{\frac{8}{15}\sqrt{\frac{2}{21}}G_{ed}^{+1}} \rho_{a_M d_{M-1}}\ (M=-2,-1,0,1) \end{aligned} \quad \textbf{(S5)}$$

$$\chi^{(7)}_{S4SM} = \sum_{M=-2,-1,0,1} \frac{\frac{2N\mu_{13}\mu_{23}\mu_{24}\mu_{14}^{3}\mu_{45}^{2}}{\varepsilon_0\hbar^7}}{\begin{array}{l}(\Gamma_{31M}+i\Delta_2)(\Gamma_{21M}+i\delta_2)(\Gamma_{41M}+i\delta_2+i\Delta_1+\frac{(\frac{\sqrt{5}}{6}G_{1M})^2(2\cos^2\theta\sin^2\theta)}{\Gamma_{21M}+i\delta_2})\\(\Gamma_{11M}+i\delta_2+i\delta_1)(\Gamma_{41M}+i\delta_2+i\delta_1+i\Delta_4)(\Gamma_{51M}+i\delta_2+i\delta_1+i\Delta_4+i\Delta_3)\\(\Gamma_{41M}-i\delta_4+i\Delta_4)\end{array}}$$

(S6)

$$\chi^{(7)}_{S4DM} = \sum_{M=-2,-1,0,1} \frac{\frac{2N\mu_{13}\mu_{23}\mu_{24}\mu_{14}^{3}\mu_{45}^{2}}{\varepsilon_0\hbar^7}}{\begin{array}{l}(\Gamma_{31M}+i\Delta_2)(\Gamma_{21M}+i\delta_2)(\Gamma_{41M}+i\delta_2+i\Delta_1+\frac{(\frac{\sqrt{5}}{6}G_{1M})^2(2\cos^2\theta\sin^2\theta)}{\Gamma_{21M}+i\delta_2})\\(\Gamma_{11M}+i\delta_2+i\delta_1)(\Gamma_{41M}+i\delta_2+i\delta_1+i\Delta_4+\frac{(\frac{2}{3}\sqrt{\frac{1}{5}}G_{4M})^2(2\cos^2\theta\sin^2\theta)}{\Gamma_{11M}+i\delta_2+i\delta_1})\\(\Gamma_{51M}+i\delta_2+i\delta_1+i\Delta_4+i\Delta_3)(\Gamma_{41M}-i\delta_4+i\Delta_4)\end{array}}$$

(S7)

$$\chi^{(7)}_{S4TM} = \sum_{M=-2,-1,0,1} \frac{\frac{2N\mu_{13}\mu_{23}\mu_{24}\mu_{14}^{3}\mu_{45}^{2}}{\varepsilon_0\hbar^7}}{\begin{array}{l}(\Gamma_{31M}+i\Delta_2)(\Gamma_{21M}+i\delta_2)(\Gamma_{41M}+i\delta_2+i\Delta_1+\frac{(\frac{\sqrt{5}}{6}G_{1M})^2(2\cos^2\theta\sin^2\theta)}{\Gamma_{21M}+i\delta_2})\\(\Gamma_{11M}+i\delta_2+i\delta_1)(\Gamma_{41M}+i\delta_2+i\delta_1+i\Delta_4+\frac{(\frac{2}{3}\sqrt{\frac{1}{5}}G_{4M})^2(2\cos^2\theta\sin^2\theta)}{\Gamma_{11M}+i\delta_2+i\delta_1})\\(\Gamma_{51M}+i\delta_2+i\delta_1+i\Delta_4+i\Delta_3+\frac{(\frac{8}{15}\sqrt{\frac{2}{21}}G_{3M})^2(2\cos^2\theta\sin^2\theta)}{\Gamma_{41M}+i\delta_2+i\delta_1+i\Delta_4})\\(\Gamma_{41M}-i\delta_4+i\Delta_4)\end{array}}$$

(S8)

Table S1. Coherent Channels Satisfying Energy Conservation

	Coherent channels	δ_1		δ_2		δ_3		δ_4	
		Resonant position	Line width	Resonant position	Line width	Resonant position	Line width	Resonant position	Line width
Single dressing	C_1	$-\Delta_4-a_+$	$\Gamma_{41M}-\Gamma_{e1M}$	a_+	Γ_{e1M}	0	0	Δ_4	$-\Gamma_{41M}$
	C_2	$-\Delta_4-a_-$	$\Gamma_{41M}-\Gamma_{e1M}$	a_-	Γ_{e1M}	0	0	Δ_4	$-\Gamma_{41M}$
	C_3	$-\Delta_3-\Delta_4-a_+$	$\Gamma_{51M}-\Gamma_{e1M}$	a_+	Γ_{e1M}	Δ_3	$-\Gamma_{51M}+\Gamma_{41M}$	Δ_4	$-\Gamma_{41M}$
	C_4	$-\Delta_3-\Delta_4-a_-$	$\Gamma_{51M}-\Gamma_{e1M}$	a_-	Γ_{e1M}	Δ_3	$-\Gamma_{51M}+\Gamma_{41M}$	Δ_4	$-\Gamma_{41M}$
Double dressing	C_5	b_+-a_+	$\Gamma_{e2M}-\Gamma_{e1M}$	a_+	Γ_{e1M}	$-b_+-\Delta_4$	$-\Gamma_{e2M}+\Gamma_{41M}$	Δ_4	$-\Gamma_{41M}$
	C_6	b_+-a_-	$\Gamma_{e2M}-\Gamma_{e1M}$	a_-	Γ_{e1M}	$-b_+-\Delta_4$	$-\Gamma_{e2M}+\Gamma_{41M}$	Δ_4	$-\Gamma_{41M}$
	C_7	b_--a_+	$\Gamma_{e2M}-\Gamma_{e1M}$	a_+	Γ_{e1M}	$-b_--\Delta_4$	$-\Gamma_{e2M}+\Gamma_{41M}$	Δ_4	$-\Gamma_{41M}$
	C_8	b_--a_-	$\Gamma_{e2M}-\Gamma_{e1M}$	a_-	Γ_{e1M}	$-b_--\Delta_4$	$-\Gamma_{e2M}+\Gamma_{41M}$	Δ_4	$-\Gamma_{41M}$
	C_9	$-\Delta_3-\Delta_4-a_+$	$\Gamma_{51M}-\Gamma_{e1M}$	a_+	Γ_{e1M}	Δ_3	$-\Gamma_{51M}+\Gamma_{41M}$	Δ_4	$-\Gamma_{41M}$
	C_{10}	$-\Delta_3-\Delta_4-a_-$	$\Gamma_{51M}-\Gamma_{e1M}$	a_-	Γ_{e1M}	Δ_3	$-\Gamma_{51M}+\Gamma_{41M}$	Δ_4	$-\Gamma_{41M}$
Triple dressing	C_{11}	b_+-a_+	$\Gamma_{e2M}-\Gamma_{e1M}$	a_+	Γ_{e1M}	$-b_+-\Delta_4$	$-\Gamma_{e2M}+\Gamma_{41M}$	Δ_4	$-\Gamma_{41M}$
	C_{12}	b_+-a_-	$\Gamma_{e2M}-\Gamma_{e1M}$	a_-	Γ_{e1M}	$-b_+-\Delta_4$	$-\Gamma_{e2M}+\Gamma_{41M}$	Δ_4	$-\Gamma_{41M}$
	C_{13}	b_--a_+	$\Gamma_{e2M}-\Gamma_{e1M}$	a_+	Γ_{e1M}	$-b_--\Delta_4$	$-\Gamma_{e2M}+\Gamma_{41M}$	Δ_4	$-\Gamma_{41M}$
	C_{14}	b_--a_-	$\Gamma_{e2M}-\Gamma_{e1M}$	a_-	Γ_{e1M}	$-b_--\Delta_4$	$-\Gamma_{e2M}+\Gamma_{41M}$	Δ_4	$-\Gamma_{41M}$
	C_{15}	c_+-a_+	$\Gamma_{e3M}-\Gamma_{e1M}$	a_+	Γ_{e1M}	$-c_+-\Delta_4$	$-\Gamma_{e3M}+\Gamma_{41M}$	Δ_4	$-\Gamma_{41M}$
	C_{16}	c_+-a_-	$\Gamma_{e3M}-\Gamma_{e1M}$	a_-	Γ_{e1M}	$-c_+-\Delta_4$	$-\Gamma_{e3M}+\Gamma_{41M}$	Δ_4	$-\Gamma_{41M}$
	C_{17}	c_--a_+	$\Gamma_{e3M}-\Gamma_{e1M}$	a_+	Γ_{e1M}	$-c_--\Delta_4$	$-\Gamma_{e3M}+\Gamma_{41M}$	Δ_4	$-\Gamma_{41M}$
	C_{18}	c_--a_-	$\Gamma_{e3M}-\Gamma_{e1M}$	a_-	Γ_{e1M}	$-c_--\Delta_4$	$-\Gamma_{e3M}+\Gamma_{41M}$	Δ_4	$-\Gamma_{41M}$

The resonant position describes the number of channels and determines how many frequency modes each photon can generate. The line width describes the full width at half maximum of the formant in the frequency domain. From Table S1, it can be seen that there are four coherent channels (C_1 - C_4) which meet the energy conservation condition $\delta_1 + \delta_2 + \delta_3 + \delta_4 = 0$ with adopting single dressing regulation. By contrast, six coherent channels (C_5 - C_{10}) are generated with adopting double dressing regulation. Further, eight coherent channels (C_{11} - C_{18}) could be constructed with utilizing triple dressing regulation.

The actual circularly polarized modes of generated photons can be assumed as $\sigma_{S1}^{+} = \sigma_{S10}^{+} + \delta\sigma_1$, $\sigma_{S2}^{+} = \sigma_{S20}^{+} + \delta\sigma_2$, $\sigma_{S3}^{-} = \sigma_{S30}^{-} + \delta\sigma_3$ and $\sigma_{S4}^{-} = \sigma_{S40}^{-} + \delta\sigma_4$, respectively. Where $\sigma_{S1}^{+} = \sigma^{+}(\omega_{S1})$, $\sigma_{S2}^{+} = \sigma^{+}(\omega_{S2})$, $\sigma_{S3}^{-} = \sigma^{-}(\omega_{S3})$, $\sigma_{S4}^{-} = \sigma^{-}(\omega_{S4})$; $\sigma_{S10}^{+} = \sigma^{+}(\varpi_{S1})$, $\sigma_{S20}^{+} = \sigma^{+}(\varpi_{S2})$, $\sigma_{S30}^{-} = \sigma^{-}(\varpi_{S3})$, $\sigma_{S40}^{-} = \sigma^{-}(\varpi_{S4})$. $\sigma_{S1}^{+} = \sigma^{+}(\omega_{S1})$ means that σ_{S1}^{+} is a function of ω_{S1}, and dependent on ω_{S1}. Other cases can be defined in the same way. σ_{S10}^{+}, σ_{S20}^{+}, σ_{S30}^{-} and σ_{S40}^{-} are the central circularly polarized modes of S1-4 photons, respectively. $\delta\sigma_{\mathrm{i}}$ is the quantum deviation of the actual circularly polarized mode of Si photon relative to its central circularly polarized mode. From the condition $\sigma_{S1}^{+} + \sigma_{S2}^{+} + \sigma_{S3}^{-} + \sigma_{S4}^{-} = \sigma_{S10}^{+} + \sigma_{S20}^{+} + \sigma_{S30}^{-} + \sigma_{S40}^{-}$ of polarization conservation, we can get $\delta\sigma_1 + \delta\sigma_2 + \delta\sigma_3 + \delta\sigma_4 = 0$.

$$
\begin{aligned}
&\left|\Psi^{1SM}_{S1\text{-}4}\right\rangle = \\
&(N_1\left|\varpi_{S1}-\Delta_4-a_+ +i\Gamma_{41M}-i\Gamma_{e1M},\varpi_{S2}+a_+ +i\Gamma_{e1M},\varpi_{S3},\varpi_{S4}+\Delta_4-i\Gamma_{41M}\right\rangle \\
&-N_2\left|\varpi_{S1}-\Delta_4-a_- +i\Gamma_{41M}-i\Gamma_{e1M},\varpi_{S2}+a_- +i\Gamma_{e1M},\varpi_{S3},\varpi_{S4}+\Delta_4-i\Gamma_{41M}\right\rangle \\
&-N_3\left|\begin{matrix}\varpi_{S1}-\Delta_3-\Delta_4-a_+ +i\Gamma_{51M}-i\Gamma_{e1M},\varpi_{S2}+a_+ +i\Gamma_{e1M}, \\ \varpi_{S3}+\Delta_3-i\Gamma_{51M}+i\Gamma_{41M},\varpi_{S4}+\Delta_4-i\Gamma_{41M}\end{matrix}\right\rangle \\
&-N_4\left|\begin{matrix}\varpi_{S1}-\Delta_3-\Delta_4-a_- +i\Gamma_{51M}-i\Gamma_{e1M},\varpi_{S2}+a_- +i\Gamma_{e1M}, \\ \varpi_{S3}+\Delta_3-i\Gamma_{51M}+i\Gamma_{41M},\varpi_{S4}+\Delta_4-i\Gamma_{41M}\end{matrix}\right\rangle) \\
&\frac{1}{2\sqrt{2}}(\left|\sigma^+_{S10}+\delta\sigma_{11},\sigma^+_{S20}+\delta\sigma_{21},\sigma^-_{S30}-\delta\sigma_{11}-\delta\sigma_{21}-\delta\sigma_4,\sigma^-_{S40}+\delta\sigma_4\right\rangle \\
&+\left|\sigma^+_{S10}+\delta\sigma_{12},\sigma^+_{S20}+\delta\sigma_{22},\sigma^-_{S30}-\delta\sigma_{12}-\delta\sigma_{22}-\delta\sigma_4,\sigma^-_{S40}+\delta\sigma_4\right\rangle \\
&+\left|\sigma^+_{S10}+\delta\sigma_{13},\sigma^+_{S20}+\delta\sigma_{21},\sigma^-_{S30}-\delta\sigma_{13}-\delta\sigma_{21}-\delta\sigma_4,\sigma^-_{S40}+\delta\sigma_4\right\rangle \\
&+\left|\sigma^+_{S10}+\delta\sigma_{14},\sigma^+_{S20}+\delta\sigma_{22},\sigma^-_{S30}-\delta\sigma_{14}-\delta\sigma_{22}-\delta\sigma_4,\sigma^-_{S40}+\delta\sigma_4\right\rangle)
\end{aligned}
\tag{S9}
$$

$$
\begin{aligned}
&\left|\Psi^{1DM}_{S1\text{-}4}\right\rangle = \\
&(N_5\left|\begin{matrix}\varpi_{S1}+b_+ -a_+ +i\Gamma_{e2M}-i\Gamma_{e1M},\varpi_{S2}+a_+ +i\Gamma_{e1M}, \\ \varpi_{S3}-b_+ -\Delta_4-i\Gamma_{e2M}+i\Gamma_{41M},\varpi_{S4}+\Delta_4-i\Gamma_{41M}\end{matrix}\right\rangle \\
&-N_6\left|\begin{matrix}\varpi_{S1}+b_+ -a_- +i\Gamma_{e2M}-i\Gamma_{e1M},\varpi_{S2}+a_- +i\Gamma_{e1M}, \\ \varpi_{S3}-b_+ -\Delta_4-i\Gamma_{e2M}+i\Gamma_{41M},\varpi_{S4}+\Delta_4-i\Gamma_{41M}\end{matrix}\right\rangle \\
&-N_7\left|\begin{matrix}\varpi_{S1}+b_- -a_+ +i\Gamma_{e2M}-i\Gamma_{e1M},\varpi_{S2}+a_+ +i\Gamma_{e1M}, \\ \varpi_{S3}-b_- -\Delta_4-i\Gamma_{e2M}+i\Gamma_{41M},\varpi_{S4}+\Delta_4-i\Gamma_{41M}\end{matrix}\right\rangle \\
&-N_8\left|\begin{matrix}\varpi_{S1}+b_- -a_- +i\Gamma_{e2M}-i\Gamma_{e1M},\varpi_{S2}+a_- +i\Gamma_{e1M}, \\ \varpi_{S3}-b_- -\Delta_4-i\Gamma_{e2M}+i\Gamma_{41M},\varpi_{S4}+\Delta_4-i\Gamma_{41M}\end{matrix}\right\rangle \\
&-N_9\left|\begin{matrix}\varpi_{S1}-\Delta_3-\Delta_4-a_+ +i\Gamma_{51M}-i\Gamma_{e1M},\varpi_{S2}+a_+ +i\Gamma_{e1M}, \\ \varpi_{S3}+\Delta_3-i\Gamma_{51M}+i\Gamma_{41M},\varpi_{S4}+\Delta_4-i\Gamma_{41M}\end{matrix}\right\rangle \\
&-N_{10}\left|\begin{matrix}\varpi_{S1}-\Delta_3-\Delta_4-a_- +i\Gamma_{51M}-i\Gamma_{e1M},\varpi_{S2}+a_- +i\Gamma_{e1M}, \\ \varpi_{S3}+\Delta_3-i\Gamma_{51M}+i\Gamma_{41M},\varpi_{S4}+\Delta_4-i\Gamma_{41M}\end{matrix}\right\rangle) \\
&\frac{1}{2\sqrt{3}}(\left|\sigma^+_{S10}+\delta\sigma_{15},\sigma^+_{S20}+\delta\sigma_{21},\sigma^-_{S30}-\delta\sigma_{15}-\delta\sigma_{21}-\delta\sigma_4,\sigma^-_{S40}+\delta\sigma_4\right\rangle \\
&+\left|\sigma^+_{S10}+\delta\sigma_{16},\sigma^+_{S20}+\delta\sigma_{22},\sigma^-_{S30}-\delta\sigma_{16}-\delta\sigma_{22}-\delta\sigma_4,\sigma^-_{S40}+\delta\sigma_4\right\rangle \\
&+\left|\sigma^+_{S10}+\delta\sigma_{17},\sigma^+_{S20}+\delta\sigma_{21},\sigma^-_{S30}-\delta\sigma_{17}-\delta\sigma_{21}-\delta\sigma_4,\sigma^-_{S40}+\delta\sigma_4\right\rangle \\
&+\left|\sigma^+_{S10}+\delta\sigma_{18},\sigma^+_{S20}+\delta\sigma_{22},\sigma^-_{S30}-\delta\sigma_{18}-\delta\sigma_{22}-\delta\sigma_4,\sigma^-_{S40}+\delta\sigma_4\right\rangle \\
&+\left|\sigma^+_{S10}+\delta\sigma_{13},\sigma^+_{S20}+\delta\sigma_{21},\sigma^-_{S30}-\delta\sigma_{13}-\delta\sigma_{21}-\delta\sigma_4,\sigma^-_{S40}+\delta\sigma_4\right\rangle \\
&+\left|\sigma^+_{S10}+\delta\sigma_{14},\sigma^+_{S20}+\delta\sigma_{22},\sigma^-_{S30}-\delta\sigma_{14}-\delta\sigma_{22}-\delta\sigma_4,\sigma^-_{S40}+\delta\sigma_4\right\rangle)
\end{aligned}
\tag{S10}
$$

$$
\begin{aligned}
&\left|\Psi_{S1\text{-}4}^{1TM}\right\rangle = \\
&(N_{11}\left|\begin{matrix}\varpi_{S1}+b_{+}-a_{+}+i\Gamma_{e2M}-i\Gamma_{e1M},\varpi_{S2}+a_{+}+i\Gamma_{e1M},\\ \varpi_{S3}-b_{+}-\Delta_{4}-i\Gamma_{e2M}+i\Gamma_{41M},\varpi_{S4}+\Delta_{4}-i\Gamma_{41M}\end{matrix}\right\rangle \\
&-N_{12}\left|\begin{matrix}\varpi_{S1}+b_{+}-a_{-}+i\Gamma_{e2M}-i\Gamma_{e1M},\varpi_{S2}+a_{-}+i\Gamma_{e1M},\\ \varpi_{S3}-b_{+}-\Delta_{4}-i\Gamma_{e2M}+i\Gamma_{41M},\varpi_{S4}+\Delta_{4}-i\Gamma_{41M}\end{matrix}\right\rangle \\
&-N_{13}\left|\begin{matrix}\varpi_{S1}+b_{-}-a_{+}+i\Gamma_{e2M}-i\Gamma_{e1M},\varpi_{S2}+a_{+}+i\Gamma_{e1M},\\ \varpi_{S3}-b_{-}-\Delta_{4}-i\Gamma_{e2M}+i\Gamma_{41M},\varpi_{S4}+\Delta_{4}-i\Gamma_{41M}\end{matrix}\right\rangle \\
&-N_{14}\left|\begin{matrix}\varpi_{S1}+b_{-}-a_{-}+i\Gamma_{e2M}-i\Gamma_{e1M},\varpi_{S2}+a_{-}+i\Gamma_{e1M},\\ \varpi_{S3}-b_{-}-\Delta_{4}-i\Gamma_{e2M}+i\Gamma_{41M},\varpi_{S4}+\Delta_{4}-i\Gamma_{41M}\end{matrix}\right\rangle \\
&-N_{15}\left|\begin{matrix}\varpi_{S1}+c_{+}-a_{+}+i\Gamma_{e3M}-i\Gamma_{e1M},\varpi_{S2}+a_{+}+i\Gamma_{e1M},\\ \varpi_{S3}-c_{+}-\Delta_{4}-i\Gamma_{e3M}+i\Gamma_{41M},\varpi_{S4}+\Delta_{4}-i\Gamma_{41M}\end{matrix}\right\rangle \\
&-N_{16}\left|\begin{matrix}\varpi_{S1}+c_{+}-a_{-}+i\Gamma_{e3M}-i\Gamma_{e1M},\varpi_{S2}+a_{-}+i\Gamma_{e1M},\\ \varpi_{S3}-c_{+}-\Delta_{4}-i\Gamma_{e3M}+i\Gamma_{41M},\varpi_{S4}+\Delta_{4}-i\Gamma_{41M}\end{matrix}\right\rangle \\
&-N_{17}\left|\begin{matrix}\varpi_{S1}+c_{-}-a_{+}+i\Gamma_{e3M}-i\Gamma_{e1M},\varpi_{S2}+a_{+}+i\Gamma_{e1M},\\ \varpi_{S3}-c_{-}-\Delta_{4}-i\Gamma_{e3M}+i\Gamma_{41M},\varpi_{S4}+\Delta_{4}-i\Gamma_{41M}\end{matrix}\right\rangle \\
&-N_{18}\left|\begin{matrix}\varpi_{S1}+c_{-}-a_{-}+i\Gamma_{e3M}-i\Gamma_{e1M},\varpi_{S2}+a_{-}+i\Gamma_{e1M},\\ \varpi_{S3}-c_{-}-\Delta_{4}-i\Gamma_{e3M}+i\Gamma_{41M},\varpi_{S4}+\Delta_{4}-i\Gamma_{41M}\end{matrix}\right\rangle) \\
&\frac{1}{4}(\left|\sigma_{S10}^{+}+\delta\sigma_{15},\sigma_{S20}^{+}+\delta\sigma_{21},\sigma_{S30}^{-}-\delta\sigma_{15}-\delta\sigma_{21}-\delta\sigma_{4},\sigma_{S40}^{-}+\delta\sigma_{4}\right\rangle \\
&+\left|\sigma_{S10}^{+}+\delta\sigma_{16},\sigma_{S20}^{+}+\delta\sigma_{22},\sigma_{S30}^{-}-\delta\sigma_{16}-\delta\sigma_{22}-\delta\sigma_{4},\sigma_{S40}^{-}+\delta\sigma_{4}\right\rangle \\
&+\left|\sigma_{S10}^{+}+\delta\sigma_{17},\sigma_{S20}^{+}+\delta\sigma_{21},\sigma_{S30}^{-}-\delta\sigma_{17}-\delta\sigma_{21}-\delta\sigma_{4},\sigma_{S40}^{-}+\delta\sigma_{4}\right\rangle \\
&+\left|\sigma_{S10}^{+}+\delta\sigma_{18},\sigma_{S20}^{+}+\delta\sigma_{22},\sigma_{S30}^{-}-\delta\sigma_{18}-\delta\sigma_{22}-\delta\sigma_{4},\sigma_{S40}^{-}+\delta\sigma_{4}\right\rangle \\
&+\left|\sigma_{S10}^{+}+\delta\sigma_{19},\sigma_{S20}^{+}+\delta\sigma_{21},\sigma_{S30}^{-}-\delta\sigma_{19}-\delta\sigma_{21}-\delta\sigma_{4},\sigma_{S40}^{-}+\delta\sigma_{4}\right\rangle \\
&+\left|\sigma_{S10}^{+}+\delta\sigma_{110},\sigma_{S20}^{+}+\delta\sigma_{22},\sigma_{S30}^{-}-\delta\sigma_{110}-\delta\sigma_{22}-\delta\sigma_{4},\sigma_{S40}^{-}+\delta\sigma_{4}\right\rangle \\
&+\left|\sigma_{S10}^{+}+\delta\sigma_{111},\sigma_{S20}^{+}+\delta\sigma_{21},\sigma_{S30}^{-}-\delta\sigma_{111}-\delta\sigma_{21}-\delta\sigma_{4},\sigma_{S40}^{-}+\delta\sigma_{4}\right\rangle \\
&+\left|\sigma_{S10}^{+}+\delta\sigma_{112},\sigma_{S20}^{+}+\delta\sigma_{22},\sigma_{S30}^{-}-\delta\sigma_{112}-\delta\sigma_{22}-\delta\sigma_{4},\sigma_{S40}^{-}+\delta\sigma_{4}\right\rangle)
\end{aligned}
\quad \textbf{(S11)}
$$

where $N_{1\text{-}4}$ satisfy $N_1^2+N_2^2+N_3^2+N_4^2=1$ and $N_1^2-N_2^2-N_3^2-N_4^2=0$ caused by destructive interference and normalization; $N_{5\text{-}10}$ satisfy $\sum_{i=5}^{10}N_i^2=1$ and $N_5^2-\sum_{i=6}^{10}N_i^2=0$; $N_{11\text{-}18}$ satisfy $\sum_{i=11}^{18}N_i^2=1$ and $N_{11}^2-\sum_{i=12}^{18}N_i^2=0$.

$$
\begin{aligned}
&B_{SM}(\tau_1,\tau_2,\tau_3)= \\
&[\frac{e^{-i(i(\Gamma_{41M}-\Gamma_{e1M})-\Delta_4-a_+)\tau_1}}{(a_- - a_+)(i(\Gamma_{41M}-\Gamma_{51M})+\Delta_3)(i(\Gamma_{41M}-\Gamma_{51M})+\Delta_3+a_- - a_+)} \\
&+\frac{e^{-i(i(\Gamma_{41M}-\Gamma_{e1M})-\Delta_4-a_-)\tau_1}}{(a_+ - a_-)(i(\Gamma_{41M}-\Gamma_{51M})+\Delta_3+a_+ - a_-)(i(\Gamma_{41M}-\Gamma_{51M})+\Delta_3)} \\
&+\frac{e^{-i(i(\Gamma_{51M}-\Gamma_{e1M})-\Delta_3-\Delta_4-a_+)\tau_1}}{(i(\Gamma_{51M}-\Gamma_{41M})-\Delta_3)(i(\Gamma_{51M}-\Gamma_{41M})-\Delta_3+a_- - a_+)(a_- - a_+)} \\
&+\frac{e^{-i(i(\Gamma_{51M}-\Gamma_{e1M})-\Delta_3-\Delta_4-a_-)\tau_1}}{(i(\Gamma_{51M}-\Gamma_{41M})-\Delta_3+a_+ - a_-)(i(\Gamma_{51M}-\Gamma_{41M})-\Delta_3)(a_+ - a_-)}] \\
&[\frac{e^{-i(i\Gamma_{e1M}+a_+)\tau_2}}{a_+ - a_-}+\frac{e^{-i(i\Gamma_{e1M}+a_-)\tau_2}}{a_- - a_+}][\frac{e^{-i*0*\tau_3}}{i(\Gamma_{51M}-\Gamma_{41M})-\Delta_3}+\frac{e^{-i(i(\Gamma_{41M}-\Gamma_{51M})+\Delta_3)\tau_3}}{i(\Gamma_{41M}-\Gamma_{51M})+\Delta_3}]
\end{aligned}
\quad \textbf{(S12)}
$$

$$
\begin{aligned}
&B_{DM}(\tau_1,\tau_2,\tau_3)= \\
&[\frac{e^{-i(i(\Gamma_{e2M}-\Gamma_{e1M})+b_+-a_+)\tau_1}}{(a_- - a_+)(b_+ - b_-)(a_- - a_+ + b_+ - b_-)(i(\Gamma_{e2M}-\Gamma_{51M})+\Delta_3+\Delta_4+b_+)(i(\Gamma_{e2M}-\Gamma_{51M})+\Delta_3+\Delta_4+a_- - a_+ + b_+)} \\
&+\frac{e^{-i(i(\Gamma_{e2M}-\Gamma_{e1M})+b_+-a_-)\tau_1}}{(a_+ - a_-)(a_+ - a_- + b_+ - b_-)(b_+ - b_-)(i(\Gamma_{e2M}-\Gamma_{51M})+\Delta_3+\Delta_4+a_+ - a_- + b_+)(i(\Gamma_{e2M}-\Gamma_{51M})+\Delta_3+\Delta_4+b_+)} \\
&+\frac{e^{-i(i(\Gamma_{e2M}-\Gamma_{e1M})+b_--a_+)\tau_1}}{(b_- - b_+)(a_- - a_+ + b_- - b_+)(a_- - a_+)(i(\Gamma_{e2M}-\Gamma_{51M})+\Delta_3+\Delta_4+b_-)(i(\Gamma_{e2M}-\Gamma_{51M})+\Delta_3+\Delta_4+a_- - a_+ + b_-)} \\
&+\frac{e^{-i(i(\Gamma_{e2M}-\Gamma_{e1M})+b_--a_-)\tau_1}}{(a_+ - a_- + b_- - b_+)(b_- - b_+)(a_+ - a_-)(i(\Gamma_{e2M}-\Gamma_{51M})+\Delta_3+\Delta_4+a_+ - a_- + b_-)(i(\Gamma_{e2M}-\Gamma_{51M})+\Delta_3+\Delta_4+b_-)} \\
&+\frac{e^{-i(i(\Gamma_{51M}-\Gamma_{e1M})-\Delta_3-\Delta_4-a_+)\tau_1}}{(i(\Gamma_{51M}-\Gamma_{e2M})-\Delta_3-\Delta_4-b_+)(i(\Gamma_{51M}-\Gamma_{e2M})-\Delta_3-\Delta_4+a_- - a_+ - b_+)(i(\Gamma_{51M}-\Gamma_{e2M})-\Delta_3-\Delta_4-b_-)(i(\Gamma_{51M}-\Gamma_{e2M})-\Delta_3-\Delta_4+a_- - a_+ - b_-)(a_- - a_+)} \\
&+\frac{e^{-i(i(\Gamma_{51M}-\Gamma_{e1M})-\Delta_3-\Delta_4-a_-)\tau_1}}{(i(\Gamma_{51M}-\Gamma_{e2M})-\Delta_3-\Delta_4+a_+ - a_- - b_+)(i(\Gamma_{51M}-\Gamma_{e2M})-\Delta_3-\Delta_4-b_+)(i(\Gamma_{51M}-\Gamma_{e2M})-\Delta_3-\Delta_4+a_+ - a_- - b_-)(i(\Gamma_{51M}-\Gamma_{e2M})-\Delta_3-\Delta_4-b_-)(a_+ - a_-)}] \\
&[\frac{e^{-i(i\Gamma_{e1M}+a_+)\tau_2}}{a_+ - a_-}+\frac{e^{-i(i\Gamma_{e1M}+a_-)\tau_2}}{a_- - a_+}][\frac{e^{-i(i(\Gamma_{41M}-\Gamma_{e2M})-b_+-\Delta_4)\tau_3}}{(b_- - b_+)(i(\Gamma_{51M}-\Gamma_{e2M})-b_+-\Delta_4-\Delta_3)} \\
&+\frac{e^{-i(i(\Gamma_{41M}-\Gamma_{e2M})-b_--\Delta_4)\tau_3}}{(b_+ - b_-)(i(\Gamma_{51M}-\Gamma_{e2M})-b_--\Delta_4-\Delta_3)} \\
&+\frac{e^{-i(i(\Gamma_{41M}-\Gamma_{51M})+\Delta_3)\tau_3}}{(i(\Gamma_{e2M}-\Gamma_{51M})+b_++\Delta_4+\Delta_3)(i(\Gamma_{e2M}-\Gamma_{51M})+b_-+\Delta_4+\Delta_3)}]
\end{aligned}
\quad \textbf{(S13)}
$$

$$
\begin{aligned}
&B_{TM}(\tau_1,\tau_2,\tau_3)= \\
&[\frac{e^{-i(i(\Gamma_{e2M}-\Gamma_{e1M})+b_+-a_+)\tau_1}}{(a_--a_+)(b_+-b_-)(a_--a_++b_+-b_-)(i(\Gamma_{e2M}-\Gamma_{e3M})+b_+-c_+)(i(\Gamma_{e2M}-\Gamma_{e3M})+a_--a_++b_+-c_+)(i(\Gamma_{e2M}-\Gamma_{e3M})+b_+-c_-)(i(\Gamma_{e2M}-\Gamma_{e3M})+a_--a_++b_+-c_-)} \\
&+\frac{e^{-i(i(\Gamma_{e2M}-\Gamma_{e1M})+b_+-a_-)\tau_1}}{(a_+-a_-)(a_+-a_-+b_+-b_-)(b_+-b_-)(i(\Gamma_{e2M}-\Gamma_{e3M})+a_+-a_-+b_+-c_+)(i(\Gamma_{e2M}-\Gamma_{e3M})+b_+-c_+)(i(\Gamma_{e2M}-\Gamma_{e3M})+a_+-a_-+b_+-c_-)(i(\Gamma_{e2M}-\Gamma_{e3M})+b_+-c_-)} \\
&+\frac{e^{-i(i(\Gamma_{e2M}-\Gamma_{e1M})+b_--a_+)\tau_1}}{(b_--b_+)(a_--a_++b_--b_+)(a_--a_+)(i(\Gamma_{e2M}-\Gamma_{e3M})+b_--c_+)(i(\Gamma_{e2M}-\Gamma_{e3M})+a_--a_++b_--c_+)(i(\Gamma_{e2M}-\Gamma_{e3M})+b_--c_-)(i(\Gamma_{e2M}-\Gamma_{e3M})+a_--a_++b_--c_-)} \\
&+\frac{e^{-i(i(\Gamma_{e2M}-\Gamma_{e1M})+b_--a_-)\tau_1}}{(a_+-a_-+b_--b_+)(b_--b_+)(a_+-a_-)(i(\Gamma_{e2M}-\Gamma_{e3M})+a_+-a_-+b_--c_+)(i(\Gamma_{e2M}-\Gamma_{e3M})+b_--c_+)(i(\Gamma_{e2M}-\Gamma_{e3M})+a_+-a_-+b_--c_-)(i(\Gamma_{e2M}-\Gamma_{e3M})+b_--c_-)} \\
&+\frac{e^{-i(i(\Gamma_{e3M}-\Gamma_{e1M})+c_+-a_+)\tau_1}}{(i(\Gamma_{e3M}-\Gamma_{e2M})+c_+-b_+)(i(\Gamma_{e3M}-\Gamma_{e2M})+a_--a_++c_+-b_+)(i(\Gamma_{e3M}-\Gamma_{e2M})+c_+-b_-)(i(\Gamma_{e3M}-\Gamma_{e2M})+a_--a_++c_+-b_-)(a_--a_+)(c_+-c_-)(a_--a_++c_+-c_-)} \\
&+\frac{e^{-i(i(\Gamma_{e3M}-\Gamma_{e1M})+c_+-a_-)\tau_1}}{(i(\Gamma_{e3M}-\Gamma_{e2M})+a_+-a_-+c_+-b_+)(i(\Gamma_{e3M}-\Gamma_{e2M})+c_+-b_+)(i(\Gamma_{e3M}-\Gamma_{e2M})+a_+-a_-+c_+-b_-)(i(\Gamma_{e3M}-\Gamma_{e2M})+c_+-b_-)(a_+-a_-)(a_+-a_-+c_+-c_-)(c_+-c_-)} \\
&+\frac{e^{-i(i(\Gamma_{e3M}-\Gamma_{e1M})+c_--a_+)\tau_1}}{(i(\Gamma_{e3M}-\Gamma_{e2M})+c_--b_+)(i(\Gamma_{e3M}-\Gamma_{e2M})+a_--a_++c_--b_+)(i(\Gamma_{e3M}-\Gamma_{e2M})+c_--b_-)(i(\Gamma_{e3M}-\Gamma_{e2M})+a_--a_++c_--b_-)(c_--c_+)(a_--a_++c_--c_+)(a_--a_+)} \\
&+\frac{e^{-i(i(\Gamma_{e3M}-\Gamma_{e1M})+c_--a_-)\tau_1}}{(i(\Gamma_{e3M}-\Gamma_{e2M})+a_+-a_-+c_--b_+)(i(\Gamma_{e3M}-\Gamma_{e2M})+c_--b_+)(i(\Gamma_{e3M}-\Gamma_{e2M})+a_+-a_-+c_--b_-)(i(\Gamma_{e3M}-\Gamma_{e2M})+c_--b_-)(a_+-a_-+c_--c_+)(c_--c_+)(a_+-a_-)}] \\
&[\frac{e^{-i(i\Gamma_{e1M}+a_+)\tau_2}}{a_+-a_-}+\frac{e^{-i(i\Gamma_{e1M}+a_-)\tau_2}}{a_--a_+}][\frac{e^{-i(i(\Gamma_{41M}-\Gamma_{e2M})-b_+-\Delta_4)\tau_3}}{(b_--b_+)(i(\Gamma_{e3M}-\Gamma_{e2M})-b_++c_+)(i(\Gamma_{e3M}-\Gamma_{e2M})-b_++c_-)} \\
&+\frac{e^{-i(i(\Gamma_{41M}-\Gamma_{e2M})-b_--\Delta_4)\tau_3}}{(b_+-b_-)(i(\Gamma_{e3M}-\Gamma_{e2M})-b_-+c_+)(i(\Gamma_{e3M}-\Gamma_{e2M})-b_-+c_-)} \\
&+\frac{e^{-i(i(\Gamma_{41M}-\Gamma_{e3M})-c_+-\Delta_4)\tau_3}}{(i(\Gamma_{e2M}-\Gamma_{e3M})+b_+-c_+)(i(\Gamma_{e2M}-\Gamma_{e3M})+b_--c_+)(c_--c_+)} \\
&+\frac{e^{-i(i(\Gamma_{41M}-\Gamma_{e3M})-c_--\Delta_4)\tau_3}}{(i(\Gamma_{e2M}-\Gamma_{e3M})+b_+-c_-)(i(\Gamma_{e2M}-\Gamma_{e3M})+b_--c_-)(c_+-c_-)}]
\end{aligned}
$$

(S14)

$$\delta_2 = \frac{i(\Gamma_{21M}+\Gamma_{41M})-\Delta_1 \pm \sqrt{[i(\Gamma_{21M}+\Gamma_{41M})-\Delta_1]^2+4(\Gamma_{21M}\Gamma_{41M}+i\Gamma_{21M}\Delta_1+\left|\frac{\sqrt{5}}{6}G_{1M}\sqrt{2\cos^2\theta\sin^2\theta}\right|^2)}}{2} \tag{S15}$$

$$\delta_1 = \begin{cases} \dfrac{i(\Gamma_{11M}+\Gamma_{41M})-\Delta_4 \pm \sqrt{[i(\Gamma_{11M}+\Gamma_{41M})-\Delta_4]^2+4(\Gamma_{11M}\Gamma_{41M}+i\Gamma_{11M}\Delta_4+\left|\frac{2}{3}\sqrt{\frac{1}{5}}G_{4M}\sqrt{2\cos^2\theta\sin^2\theta}\right|^2)}}{2}-\delta_2, \\ \dfrac{i(\Gamma_{41M}+\Gamma_{51M})-2\Delta_4-\Delta_3 \pm \sqrt{[i(\Gamma_{41M}+\Gamma_{51M})-2\Delta_4-\Delta_3]^2+4[\Gamma_{41M}\Gamma_{51M}+i\Gamma_{41M}(\Delta_4+\Delta_3)+i\Gamma_{51M}\Delta_4-\Delta_4(\Delta_4+\Delta_3)+\left|\frac{8}{15}\sqrt{\frac{2}{21}}G_{3M}\sqrt{2\cos^2\theta\sin^2\theta}\right|^2]}}{2}-\delta_2 \end{cases} \tag{S16}$$

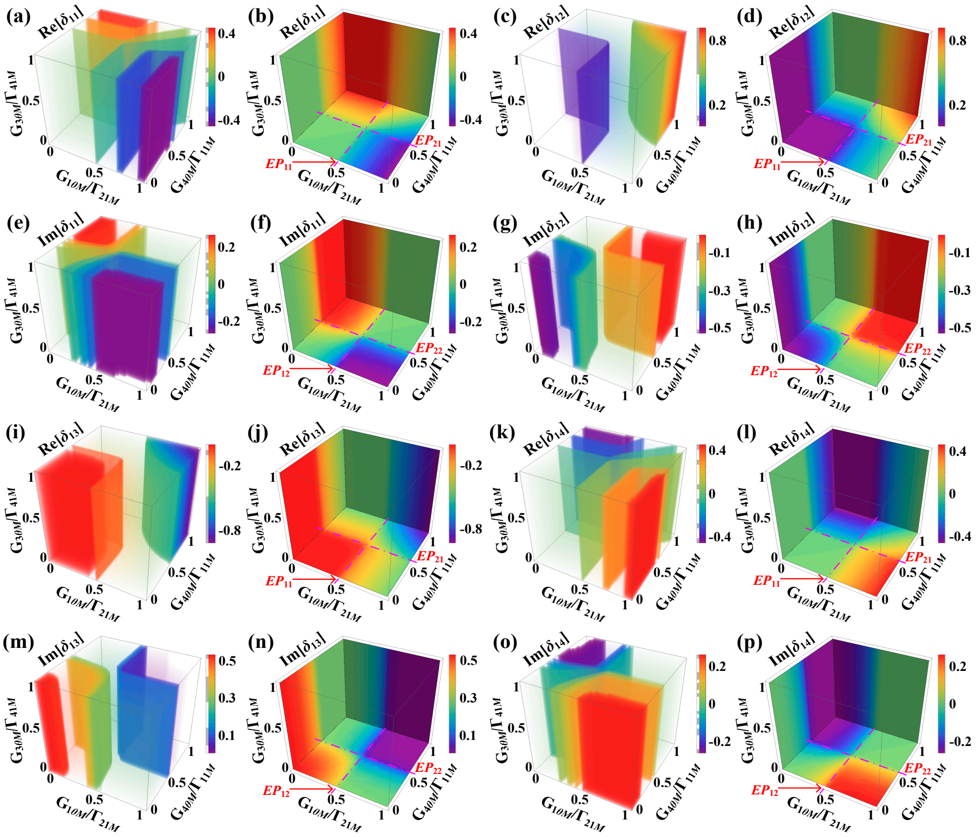

Fig. S1. Real and imaginary part of the first to fourth eigenstates of δ_1, in which δ_1 represents the multi-eigenenergy nature of generated quadphoton. $G_{1\theta M}=\sqrt{5\cos^2\theta\sin^2\theta/18}G_{1M}$, $G_{4\theta M}=\sqrt{8\cos^2\theta\sin^2\theta/45}G_{4M}$, $G_{3\theta M}=\sqrt{256\cos^2\theta\sin^2\theta/4725}G_{3M}$. (a), (c), (i) and (k) Density plot of real part of the first to fourth eigenstates, respectively. (e), (g), (m) and (o) Density plot of imaginary part of the first to fourth eigenstates, respectively. At $[G_{1\theta M}, G_{4\theta M}, G_{3\theta M}]=[G_{1\theta M}, G_{4\theta M}, 0]$, $[0, G_{4\theta M}, G_{3\theta M}]$ and $[G_{1\theta M}, \Gamma_{11M}, G_{3\theta M}]$, (b), (d), (f), (h), (j), (l), (n) and (p) is the slice plot of (a), (c), (e), (g), (i), (k), (m) and (o), respectively. Two pairs of eigenstates degenerate into one eigenstate at EP_{11} / EP_{12} ($G_{1\theta M}=0.5\Gamma_{21M}$) and EP_{21} / EP_{22} ($G_{4\theta M}=0.5\Gamma_{11M}$), respectively.

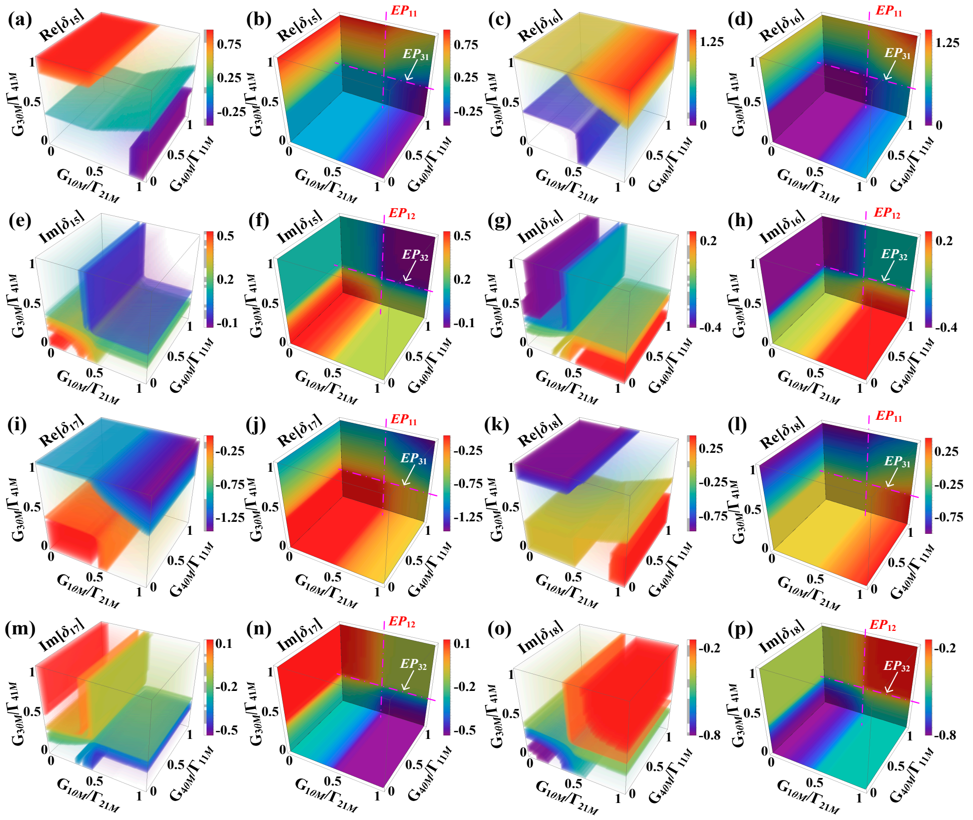

Fig. S2. Real and imaginary part of the fifth to eighth eigenstates of δ_1. Similar to Fig. S1, but another pair of eigenstates degenerate into one eigenstate at EP_{31} / EP_{32} ($G_{3\theta M}=0.38\Gamma_{41M}$).

Table S2. Real and imaginary part of coherent channels with triple dressing

Coherent channels		δ_1		δ_2		δ_3		δ_4	
		Resonant position	Line width	Resonant position	Line width	Resonant position	Line width	Resonant position	Line width
Real part	Re$C_{1'}$	b_{+}-a_{+}	Γ_{e5+M}-Γ_{e4+M}	a_{+}	Γ_{e4+M}	-b_{+}-Δ_4	-Γ_{e5+M}+Γ_{41M}	Δ_4	-Γ_{41M}
	Re$C_{2'}$	b_{+}-a_{-}	Γ_{e5+M}-Γ_{e4-M}	a_{-}	Γ_{e4-M}	-b_{+}-Δ_4	-Γ_{e5+M}+Γ_{41M}	Δ_4	-Γ_{41M}
	Re$C_{3'}$	b_{-}-a_{+}	Γ_{e5-M}-Γ_{e4+M}	a_{+}	Γ_{e4+M}	-b_{-}-Δ_4	-Γ_{e5-M}+Γ_{41M}	Δ_4	-Γ_{41M}
	Re$C_{4'}$	b_{-}-a_{-}	Γ_{e5-M}-Γ_{e4-M}	a_{-}	Γ_{e4-M}	-b_{-}-Δ_4	-Γ_{e5-M}+Γ_{41M}	Δ_4	-Γ_{41M}
	Re$C_{5'}$	c_{+}-a_{+}	Γ_{e6+M}-Γ_{e4+M}	a_{+}	Γ_{e4+M}	-c_{+}-Δ_4	-Γ_{e6+M}+Γ_{41M}	Δ_4	-Γ_{41M}
	Re$C_{6'}$	c_{+}-a_{-}	Γ_{e6+M}-Γ_{e4-M}	a_{-}	Γ_{e4-M}	-c_{+}-Δ_4	-Γ_{e6+M}+Γ_{41M}	Δ_4	-Γ_{41M}
	Re$C_{7'}$	c_{-}-a_{+}	Γ_{e6-M}-Γ_{e4+M}	a_{+}	Γ_{e4+M}	-c_{-}-Δ_4	-Γ_{e6-M}+Γ_{41M}	Δ_4	-Γ_{41M}
	Re$C_{8'}$	c_{-}-a_{-}	Γ_{e6-M}-Γ_{e4-M}	a_{-}	Γ_{e4-M}	-c_{-}-Δ_4	-Γ_{e6-M}+Γ_{41M}	Δ_4	-Γ_{41M}
EP	ReC_{EP41}	(Δ_1-Δ_4)/2	Γ_{e2M}-Γ_{e1M}	-Δ_1/2	Γ_{e1M}	-Δ_4/2	Γ_{41M}-Γ_{e2M}	Δ_4	-Γ_{41M}
	ReC_{EP51}	(Δ_1-Δ_3-2Δ_4)/2	Γ_{e3M}-Γ_{e1M}	-Δ_1/2	Γ_{e1M}	Δ_3/2	Γ_{41M}-Γ_{e3M}	Δ_4	-Γ_{41M}
	ImC_{EP42}	Γ_{e2M}-Γ_{e1M}	(Δ_1-Δ_4)/2	$\Gamma_{e1\mathrm{M}}$	-Δ_1/2	Γ_{41M}-Γ_{e2M}	-Δ_4/2	-Γ_{41M}	Δ_4
	ImC_{EP52}	Γ_{e3M}-Γ_{e1M}	(Δ_1-Δ_3-2Δ_4)/2	$\Gamma_{e1\mathrm{M}}$	-Δ_1/2	Γ_{41M}-Γ_{e3M}	Δ_3/2	-Γ_{41M}	Δ_4
Imaginary part	Im$C_{1'}$	e_{+}-d_{+}	Δ_{e2+}-Δ_{e1+}	d_{+}	Δ_{e1+}	Γ_{41M}-e_{+}	-Δ_4-Δ_{e2+}	-Γ_{41M}	Δ_4
	Im$C_{2'}$	e_{+}-d_{-}	Δ_{e2+}-Δ_{e1-}	d_{-}	Δ_{e1-}	Γ_{41M}-e_{+}	-Δ_4-Δ_{e2+}	-Γ_{41M}	Δ_4
	Im$C_{3'}$	e_{-}-d_{+}	Δ_{e2-}-Δ_{e1+}	d_{+}	Δ_{e1+}	Γ_{41M}-e_{-}	-Δ_4-Δ_{e2-}	-Γ_{41M}	Δ_4
	Im$C_{4'}$	e_{-}-d_{-}	Δ_{e2-}-Δ_{e1-}	d_{-}	Δ_{e1-}	Γ_{41M}-e_{-}	-Δ_4-Δ_{e2-}	-Γ_{41M}	Δ_4
	Im$C_{5'}$	f_{+}-d_{+}	Δ_{e3+}-Δ_{e1+}	d_{+}	Δ_{e1+}	Γ_{41M}-f_{+}	-Δ_4-Δ_{e3+}	-Γ_{41M}	Δ_4
	Im$C_{6'}$	f_{+}-d_{-}	Δ_{e3+}-Δ_{e1-}	d_{-}	Δ_{e1-}	Γ_{41M}-f_{+}	-Δ_4-Δ_{e3+}	-Γ_{41M}	Δ_4
	Im$C_{7'}$	f_{-}-d_{+}	Δ_{e3-}-Δ_{e1+}	d_{+}	Δ_{e1+}	Γ_{41M}-f_{-}	-Δ_4-Δ_{e3-}	-Γ_{41M}	Δ_4
	Im$C_{8'}$	f_{-}-d_{-}	Δ_{e3-}-Δ_{e1-}	d_{-}	Δ_{e1-}	Γ_{41M}-f_{-}	-Δ_4-Δ_{e3-}	-Γ_{41M}	Δ_4

For the real part of coherent channels, the actual polarized modes are assumed as $\sigma'^{+}_{S1} = \sigma'^{+}_{S10} + \delta'\sigma_1, \sigma'^{+}_{S2} = \sigma'^{+}_{S20} + \delta'\sigma_2, \sigma'^{-}_{S3} = \sigma'^{-}_{S30} + \delta'\sigma_3$ and $\sigma'^{-}_{S4} = \sigma'^{-}_{S40} + \delta'\sigma_4$. $\sigma'^{+}_{S10}, \sigma'^{+}_{S20}, \sigma'^{-}_{S30}$ and σ'^{-}_{S40} are the central polarized modes. $\delta'\sigma_{\mathrm{i}}$ is the quantum deviation of the polarized mode of Si photon. $\sigma'^{+}_{S1} = \sigma'^{+}(\omega'_{S1}), \sigma'^{+}_{S2} = \sigma'^{+}(\omega'_{S2}), \sigma'^{-}_{S3} = \sigma'^{-}(\omega'_{S3}), \sigma'^{-}_{S4} = \sigma'^{-}(\omega'_{S4}); \sigma'^{+}_{S10} = \sigma'^{+}(\varpi'_{S1}),$ $\sigma'^{+}_{S20} = \sigma'^{+}(\varpi'_{S2}), \sigma'^{-}_{S30} = \sigma'^{-}(\varpi'_{S3}), \sigma'^{-}_{S40} = \sigma'^{-}(\varpi'_{S4})$. $\sigma'^{+}_{S1} = \sigma'^{+}(\omega'_{S1})$ means that σ'^{+}_{S1} is a function of ω'_{S1}, and dependent on ω'_{S1}. Other cases can be defined in the same way. $\omega'_{S\mathrm{i}}$ is assumed as the actual frequency and expressed as $\omega'_{S\mathrm{i}} = \varpi'_{S\mathrm{i}} + \delta_{\mathrm{i}}$, where $\varpi'_{S\mathrm{i}}$ is assumed as the central frequency. In above physical parameters of the real part of coherent channels, the character $'$ is replaced with $''$ to represent physical parameters of the imaginary part of coherent channels, and corresponding parameters are then similarly defined.

Polarization-based time-frequency entanglement states for the real and imaginary part of coherent channels can be modelled as Eqs. (S17)-(S20), respectively.

$$
\begin{aligned}
&\left|\operatorname{Re}\Psi_{S1\text{-}4}^{1T'M}\right\rangle = \\
&(N_{1'}\left|\begin{matrix}\varpi'_{S1}+b_{+}-a_{+}+i\Gamma_{e5+M}-i\Gamma_{e4+M},\varpi'_{S2}+a_{+}+i\Gamma_{e4+M}, \\ \varpi'_{S3}-b_{+}-\Delta_{4}-i\Gamma_{e5+M}+i\Gamma_{41M},\varpi'_{S4}+\Delta_{4}-i\Gamma_{41M}\end{matrix}\right\rangle \\
&-N_{2'}\left|\begin{matrix}\varpi'_{S1}+b_{+}-a_{-}+i\Gamma_{e5+M}-i\Gamma_{e4\text{-}M},\varpi'_{S2}+a_{-}+i\Gamma_{e4\text{-}M}, \\ \varpi'_{S3}-b_{+}-\Delta_{4}-i\Gamma_{e5+M}+i\Gamma_{41M},\varpi'_{S4}+\Delta_{4}-i\Gamma_{41M}\end{matrix}\right\rangle \\
&-N_{3'}\left|\begin{matrix}\varpi'_{S1}+b_{-}-a_{+}+i\Gamma_{e5\text{-}M}-i\Gamma_{e4+M},\varpi'_{S2}+a_{+}+i\Gamma_{e4+M}, \\ \varpi'_{S3}-b_{-}-\Delta_{4}-i\Gamma_{e5\text{-}M}+i\Gamma_{41M},\varpi'_{S4}+\Delta_{4}-i\Gamma_{41M}\end{matrix}\right\rangle \\
&-N_{4'}\left|\begin{matrix}\varpi'_{S1}+b_{-}-a_{-}+i\Gamma_{e5\text{-}M}-i\Gamma_{e4\text{-}M},\varpi'_{S2}+a_{-}+i\Gamma_{e4\text{-}M}, \\ \varpi'_{S3}-b_{-}-\Delta_{4}-i\Gamma_{e5\text{-}M}+i\Gamma_{41M},\varpi'_{S4}+\Delta_{4}-i\Gamma_{41M}\end{matrix}\right\rangle \\
&-N_{5'}\left|\begin{matrix}\varpi'_{S1}+c_{+}-a_{+}+i\Gamma_{e6+M}-i\Gamma_{e4+M},\varpi'_{S2}+a_{+}+i\Gamma_{e4+M}, \\ \varpi'_{S3}-c_{+}-\Delta_{4}-i\Gamma_{e6+M}+i\Gamma_{41M},\varpi'_{S4}+\Delta_{4}-i\Gamma_{41M}\end{matrix}\right\rangle \\
&-N_{6'}\left|\begin{matrix}\varpi'_{S1}+c_{+}-a_{-}+i\Gamma_{e6+M}-i\Gamma_{e4\text{-}M},\varpi'_{S2}+a_{-}+i\Gamma_{e4\text{-}M}, \\ \varpi'_{S3}-c_{+}-\Delta_{4}-i\Gamma_{e6+M}+i\Gamma_{41M},\varpi'_{S4}+\Delta_{4}-i\Gamma_{41M}\end{matrix}\right\rangle \\
&-N_{7'}\left|\begin{matrix}\varpi'_{S1}+c_{-}-a_{+}+i\Gamma_{e6\text{-}M}-i\Gamma_{e4+M},\varpi'_{S2}+a_{+}+i\Gamma_{e4+M}, \\ \varpi'_{S3}-c_{-}-\Delta_{4}-i\Gamma_{e6\text{-}M}+i\Gamma_{41M},\varpi'_{S4}+\Delta_{4}-i\Gamma_{41M}\end{matrix}\right\rangle \\
&-N_{8'}\left|\begin{matrix}\varpi'_{S1}+c_{-}-a_{-}+i\Gamma_{e6\text{-}M}-i\Gamma_{e4\text{-}M},\varpi'_{S2}+a_{-}+i\Gamma_{e4\text{-}M}, \\ \varpi'_{S3}-c_{-}-\Delta_{4}-i\Gamma_{e6\text{-}M}+i\Gamma_{41M},\varpi'_{S4}+\Delta_{4}-i\Gamma_{41M}\end{matrix}\right\rangle) \\
&\frac{1}{4}(\left|\sigma'^{+}_{S10}+\delta'\sigma_{11},\sigma'^{+}_{S20}+\delta'\sigma_{21},\sigma'^{-}_{S30}-\delta'\sigma_{11}-\delta'\sigma_{21}-\delta'\sigma_{4},\sigma'^{-}_{S40}+\delta'\sigma_{4}\right\rangle \\
&+\left|\sigma'^{+}_{S10}+\delta'\sigma_{12},\sigma'^{+}_{S20}+\delta'\sigma_{22},\sigma'^{-}_{S30}-\delta'\sigma_{12}-\delta'\sigma_{22}-\delta'\sigma_{4},\sigma'^{-}_{S40}+\delta'\sigma_{4}\right\rangle \\
&+\left|\sigma'^{+}_{S10}+\delta'\sigma_{13},\sigma'^{+}_{S20}+\delta'\sigma_{21},\sigma'^{-}_{S30}-\delta'\sigma_{13}-\delta'\sigma_{21}-\delta'\sigma_{4},\sigma'^{-}_{S40}+\delta'\sigma_{4}\right\rangle \\
&+\left|\sigma'^{+}_{S10}+\delta'\sigma_{14},\sigma'^{+}_{S20}+\delta'\sigma_{22},\sigma'^{-}_{S30}-\delta'\sigma_{14}-\delta'\sigma_{22}-\delta'\sigma_{4},\sigma'^{-}_{S40}+\delta'\sigma_{4}\right\rangle \\
&+\left|\sigma'^{+}_{S10}+\delta'\sigma_{15},\sigma'^{+}_{S20}+\delta'\sigma_{21},\sigma'^{-}_{S30}-\delta'\sigma_{15}-\delta'\sigma_{21}-\delta'\sigma_{4},\sigma'^{-}_{S40}+\delta'\sigma_{4}\right\rangle \\
&+\left|\sigma'^{+}_{S10}+\delta'\sigma_{16},\sigma'^{+}_{S20}+\delta'\sigma_{22},\sigma'^{-}_{S30}-\delta'\sigma_{16}-\delta'\sigma_{22}-\delta'\sigma_{4},\sigma'^{-}_{S40}+\delta'\sigma_{4}\right\rangle \\
&+\left|\sigma'^{+}_{S10}+\delta'\sigma_{17},\sigma'^{+}_{S20}+\delta'\sigma_{21},\sigma'^{-}_{S30}-\delta'\sigma_{17}-\delta'\sigma_{21}-\delta'\sigma_{4},\sigma'^{-}_{S40}+\delta'\sigma_{4}\right\rangle \\
&+\left|\sigma'^{+}_{S10}+\delta'\sigma_{18},\sigma'^{+}_{S20}+\delta'\sigma_{22},\sigma'^{-}_{S30}-\delta'\sigma_{18}-\delta'\sigma_{22}-\delta'\sigma_{4},\sigma'^{-}_{S40}+\delta'\sigma_{4}\right\rangle)
\end{aligned}
\tag{S17}
$$

$$\left|\mathrm{Re}\,\Psi^{1T'M}_{S1\text{-}4;EP_{41,51}}\right\rangle =$$

$$(N_{9'}\left|\begin{matrix}\varpi'_{S1;EP_{41}}+(\Delta_1-\Delta_4)/2+i\Gamma_{e2M}-i\Gamma_{e1M},\varpi'_{S2;EP_{41}}-\Delta_1/2+i\Gamma_{e1M},\\ \varpi'_{S3;EP_{41}}-\Delta_4/2+i\Gamma_{41M}-i\Gamma_{e2M},\varpi'_{S4;EP_{41}}+\Delta_4-i\Gamma_{41M}\end{matrix}\right\rangle$$

$$-N_{10'}\left|\begin{matrix}\varpi'_{S1;EP_{51}}+(\Delta_1-\Delta_3-2\Delta_4)/2+i\Gamma_{e3M}-i\Gamma_{e1M},\varpi'_{S2;EP_{51}}-\Delta_1/2+i\Gamma_{e1M},\\ \varpi'_{S3;EP_{51}}+\Delta_3/2+i\Gamma_{41M}-i\Gamma_{e3M},\varpi'_{S4;EP_{51}}+\Delta_4-i\Gamma_{41M}\end{matrix}\right\rangle) \quad \textbf{(S18)}$$

$$\frac{1}{2}(\left|\begin{matrix}\sigma'^{+}_{S10;EP_{41}}+\delta'\sigma_{11;EP_{41}},\sigma'^{+}_{S20;EP_{41}}+\delta'\sigma_{2;EP_{41}},\sigma'^{-}_{S30;EP_{41}}\\ -\delta'\sigma_{11;EP_{41}}-\delta'\sigma_{2;EP_{41}}-\delta'\sigma_{4;EP_{41}},\sigma'^{-}_{S40;EP_{41}}+\delta'\sigma_{4;EP_{41}}\end{matrix}\right\rangle$$

$$+\left|\begin{matrix}\sigma'^{+}_{S10;EP_{51}}+\delta'\sigma_{12;EP_{51}},\sigma'^{+}_{S20;EP_{51}}+\delta'\sigma_{2;EP_{51}},\sigma'^{-}_{S30;EP_{51}}\\ -\delta'\sigma_{12;EP_{51}}-\delta'\sigma_{2;EP_{51}}-\delta'\sigma_{4;EP_{51}},\sigma'^{-}_{S40;EP_{51}}+\delta'\sigma_{4;EP_{51}}\end{matrix}\right\rangle)$$

$$\left|\mathrm{Im}\,\Psi^{1T'M}_{S1\text{-}4;EP_{42,52}}\right\rangle =$$

$$(N_{11'}\left|\begin{matrix}\varpi''_{S1;EP_{42}}+\Gamma_{e2M}-\Gamma_{e1M}+i(\Delta_1-\Delta_4)/2,\varpi''_{S2;EP_{42}}+\Gamma_{e1M}-i\Delta_1/2,\\ \varpi''_{S3;EP_{42}}+\Gamma_{41M}-\Gamma_{e2M}-i\Delta_4/2,\varpi''_{S4;EP_{42}}-\Gamma_{41M}+i\Delta_4\end{matrix}\right\rangle$$

$$-N_{12'}\left|\begin{matrix}\varpi''_{S1;EP_{52}}+\Gamma_{e3M}-\Gamma_{e1M}+i(\Delta_1-\Delta_3-2\Delta_4)/2,\varpi''_{S2;EP_{52}}+\Gamma_{e1M}-i\Delta_1/2,\\ \varpi''_{S3;EP_{52}}+\Gamma_{41M}-\Gamma_{e3M}+i\Delta_3/2,\varpi''_{S4;EP_{52}}-\Gamma_{41M}+i\Delta_4\end{matrix}\right\rangle) \quad \textbf{(S19)}$$

$$\frac{1}{2}(\left|\begin{matrix}\sigma''^{+}_{S10;EP_{42}}+\delta''\sigma_{11;EP_{42}},\sigma''^{+}_{S20;EP_{42}}+\delta''\sigma_{2;EP_{42}},\sigma''^{-}_{S30;EP_{42}}\\ -\delta''\sigma_{11;EP_{42}}-\delta''\sigma_{2;EP_{42}}-\delta''\sigma_{4;EP_{42}},\sigma''^{-}_{S40;EP_{42}}+\delta''\sigma_{4;EP_{42}}\end{matrix}\right\rangle$$

$$+\left|\begin{matrix}\sigma''^{+}_{S10;EP_{52}}+\delta''\sigma_{12;EP_{52}},\sigma''^{+}_{S20;EP_{52}}+\delta''\sigma_{2;EP_{52}},\sigma''^{-}_{S30;EP_{52}}\\ -\delta''\sigma_{12;EP_{52}}-\delta''\sigma_{2;EP_{52}}-\delta''\sigma_{4;EP_{52}},\sigma''^{-}_{S40;EP_{52}}+\delta''\sigma_{4;EP_{52}}\end{matrix}\right\rangle)$$

$$
\begin{aligned}
&\left|\mathrm{Im}\,\Psi^{1T'M}_{S1\text{-}4}\right\rangle = \\
&(N_{13'}\left|\begin{aligned}&\varpi''_{S1}+e_{+}-d_{+}+i\Delta_{e2+}-i\Delta_{e1+},\varpi''_{S2}+d_{+}+i\Delta_{e1+},\\&\varpi''_{S3}+\Gamma_{41M}-e_{+}-i\Delta_{4}-i\Delta_{e2+},\varpi''_{S4}-\Gamma_{41M}+i\Delta_{4}\end{aligned}\right\rangle \\
&-N_{14'}\left|\begin{aligned}&\varpi''_{S1}+e_{+}-d_{-}+i\Delta_{e2+}-i\Delta_{e1-},\varpi''_{S2}+d_{-}+i\Delta_{e1-},\\&\varpi''_{S3}+\Gamma_{41M}-e_{+}-i\Delta_{4}-i\Delta_{e2+},\varpi''_{S4}-\Gamma_{41M}+i\Delta_{4}\end{aligned}\right\rangle \\
&-N_{15'}\left|\begin{aligned}&\varpi''_{S1}+e_{-}-d_{+}+i\Delta_{e2-}-i\Delta_{e1+},\varpi''_{S2}+d_{+}+i\Delta_{e1+},\\&\varpi''_{S3}+\Gamma_{41M}-e_{-}-i\Delta_{4}-i\Delta_{e2-},\varpi''_{S4}-\Gamma_{41M}+i\Delta_{4}\end{aligned}\right\rangle \\
&-N_{16'}\left|\begin{aligned}&\varpi''_{S1}+e_{-}-d_{-}+i\Delta_{e2-}-i\Delta_{e1-},\varpi''_{S2}+d_{-}+i\Delta_{e1-},\\&\varpi''_{S3}+\Gamma_{41M}-e_{-}-i\Delta_{4}-i\Delta_{e2-},\varpi''_{S4}-\Gamma_{41M}+i\Delta_{4}\end{aligned}\right\rangle \\
&-N_{17'}\left|\begin{aligned}&\varpi''_{S1}+f_{+}-d_{+}+i\Delta_{e3+}-i\Delta_{e1+},\varpi''_{S2}+d_{+}+i\Delta_{e1+},\\&\varpi''_{S3}+\Gamma_{41M}-f_{+}-i\Delta_{4}-i\Delta_{e3+},\varpi''_{S4}-\Gamma_{41M}+i\Delta_{4}\end{aligned}\right\rangle \\
&-N_{18'}\left|\begin{aligned}&\varpi''_{S1}+f_{+}-d_{-}+i\Delta_{e3+}-i\Delta_{e1-},\varpi''_{S2}+d_{-}+i\Delta_{e1-},\\&\varpi''_{S3}+\Gamma_{41M}-f_{+}-i\Delta_{4}-i\Delta_{e3+},\varpi''_{S4}-\Gamma_{41M}+i\Delta_{4}\end{aligned}\right\rangle \\
&-N_{19'}\left|\begin{aligned}&\varpi''_{S1}+f_{-}-d_{+}+i\Delta_{e3-}-i\Delta_{e1+},\varpi''_{S2}+d_{+}+i\Delta_{e1+},\\&\varpi''_{S3}+\Gamma_{41M}-f_{-}-i\Delta_{4}-i\Delta_{e3-},\varpi''_{S4}-\Gamma_{41M}+i\Delta_{4}\end{aligned}\right\rangle \\
&-N_{20'}\left|\begin{aligned}&\varpi''_{S1}+f_{-}-d_{-}+i\Delta_{e3-}-i\Delta_{e1-},\varpi''_{S2}+d_{-}+i\Delta_{e1-},\\&\varpi''_{S3}+\Gamma_{41M}-f_{-}-i\Delta_{4}-i\Delta_{e3-},\varpi''_{S4}-\Gamma_{41M}+i\Delta_{4}\end{aligned}\right\rangle) \\
&\frac{1}{4}(\left|\sigma''^{+}_{S10}+\delta''\sigma_{11},\sigma''^{+}_{S20}+\delta''\sigma_{21},\sigma''^{-}_{S30}-\delta''\sigma_{11}-\delta''\sigma_{21}-\delta''\sigma_{4},\sigma''^{-}_{S40}+\delta''\sigma_{4}\right\rangle \\
&+\left|\sigma''^{+}_{S10}+\delta''\sigma_{12},\sigma''^{+}_{S20}+\delta''\sigma_{22},\sigma''^{-}_{S30}-\delta''\sigma_{12}-\delta''\sigma_{22}-\delta''\sigma_{4},\sigma''^{-}_{S40}+\delta''\sigma_{4}\right\rangle \\
&+\left|\sigma''^{+}_{S10}+\delta''\sigma_{13},\sigma''^{+}_{S20}+\delta''\sigma_{21},\sigma''^{-}_{S30}-\delta''\sigma_{13}-\delta''\sigma_{21}-\delta''\sigma_{4},\sigma''^{-}_{S40}+\delta''\sigma_{4}\right\rangle \\
&+\left|\sigma''^{+}_{S10}+\delta''\sigma_{14},\sigma''^{+}_{S20}+\delta''\sigma_{22},\sigma''^{-}_{S30}-\delta''\sigma_{14}-\delta''\sigma_{22}-\delta''\sigma_{4},\sigma''^{-}_{S40}+\delta''\sigma_{4}\right\rangle \\
&+\left|\sigma''^{+}_{S10}+\delta''\sigma_{15},\sigma''^{+}_{S20}+\delta''\sigma_{21},\sigma''^{-}_{S30}-\delta''\sigma_{15}-\delta''\sigma_{21}-\delta''\sigma_{4},\sigma''^{-}_{S40}+\delta''\sigma_{4}\right\rangle \\
&+\left|\sigma''^{+}_{S10}+\delta''\sigma_{16},\sigma''^{+}_{S20}+\delta''\sigma_{22},\sigma''^{-}_{S30}-\delta''\sigma_{16}-\delta''\sigma_{22}-\delta''\sigma_{4},\sigma''^{-}_{S40}+\delta''\sigma_{4}\right\rangle \\
&+\left|\sigma''^{+}_{S10}+\delta''\sigma_{17},\sigma''^{+}_{S20}+\delta''\sigma_{21},\sigma''^{-}_{S30}-\delta''\sigma_{17}-\delta''\sigma_{21}-\delta''\sigma_{4},\sigma''^{-}_{S40}+\delta''\sigma_{4}\right\rangle \\
&+\left|\sigma''^{+}_{S10}+\delta''\sigma_{18},\sigma''^{+}_{S20}+\delta''\sigma_{22},\sigma''^{-}_{S30}-\delta''\sigma_{18}-\delta''\sigma_{22}-\delta''\sigma_{4},\sigma''^{-}_{S40}+\delta''\sigma_{4}\right\rangle)
\end{aligned}
\tag{S20}
$$

where $N_{1'\text{-}8'}$ satisfy $\sum_{\mathrm{i}=1'}^{8'} N_{\mathrm{i}}^2 = 1$ and $N_{1'}^2 - \sum_{\mathrm{i}=2'}^{8'} N_{\mathrm{i}}^2 = 0$; $N_{9',10'}$ satisfy $N_{9'}^2 + N_{10'}^2 = 1$ and $N_{9'}^2 - N_{10'}^2 = 0$; $N_{11',12'}$ satisfy $N_{11'}^2 + N_{12'}^2 = 1$ and $N_{11'}^2 - N_{12'}^2 = 0$; $N_{13'\text{-}20'}$ satisfy $\sum_{\mathrm{i}=13'}^{20'} N_{\mathrm{i}}^2 = 1$ and $N_{13'}^2 - \sum_{\mathrm{i}=14'}^{20'} N_{\mathrm{i}}^2 = 0$.
